# A Biomimetic Norepinephrine‐Loaded Aligned Mineralized Collagen Scaffold for Coordinated Neurovascular, Osteogenic, and Immunomodulatory Repair of Critical‐Sized Bone Defects

**DOI:** 10.1002/advs.202518807

**Published:** 2025-11-21

**Authors:** Zhengyun Ren, Zhaojun Wu, Anhang Wu, Hui Zhang, Jiachen Lu, Jiahao Zhang, Jie Weng, Jinhua Zhang, Song Chen, Huan Tan, Tailin Guo

**Affiliations:** ^1^ College of Medicine Southwest Jiaotong University Chengdu 610031 China; ^2^ Key Laboratory of Advanced Technologies of Materials Ministry of Education School of Materials Science and Engineering Southwest Jiaotong University Chengdu 610031 China; ^3^ Obesity and Metabolism Medicine‐Engineering Integration Laboratory Department of General Surgery The Third People's Hospital of Chengdu Affiliated Hospital of Southwest Jiaotong University Chengdu 610031 China; ^4^ Department of Orthopaedics The General Hospital of Western Theater Command Chengdu 610083 China

**Keywords:** angiogenesis, immunomodulation, mineralized collagen scaffold, neuroregeneration, norepinephrine delivery, osteogenesis

## Abstract

Effective regeneration of critical‐sized bone defects requires integrated coordination among osteogenesis, neurogenesis, angiogenesis, and immune modulation—yet most biomaterials fail to address these dimensions simultaneously. Here, a norepinephrine‐loaded, biomimetic mineralized electrocompacted collagen scaffold (NE‐MEC) is developed that integrates structural anisotropy and sustained biochemical activity to promote integrated bone repair. Fabricated through isoelectric focusing, mechanical stretching, and amorphous calcium phosphate mineralization, NE‐MEC mimics the composition and ordered alignment of native bone and preserves the characteristic D‐periodicity of collagen fibrils. The mineralized electrocompacted collagen alone promotes osteogenic differentiation of rat bone marrow mesenchymal stem cells. Upon norepinephrine incorporation, this osteoinductive effect is further enhanced, accompanied by early‐stage upregulation of nerve growth factor, which supporting peripheral nerve repair and inducing calcitonin gene‐related peptide (CGRP) production. In turn, CGRP enhances both osteogenic differentiation and neovascularization. Meanwhile, NE‐MEC also stimulates vascular endothelial growth factor A expression in the regenerating bone tissue and modulates macrophage polarization. Together, these effects establish a regenerative circuit that orchestrates osteogenic, neurogenic, angiogenic, and immunomodulatory processes, offering a promising biomaterial platform for synergistic skeletal repair.

## Graphical abstract

Inspired by the composition and structure of native bone tissue and its complex interplay of biological signals, a norepinephrine‐loaded biomimetic mineralized electrocompacted collagen scaffold (NE‐MEC) is developed capable of simultaneously supporting osteogenesis, neural repair, angiogenesis, and immune modulation. This NE‐MEC scaffold combines biomimetic alignment, bone‐mimicking composition, and sustained biochemical signaling to engage multiple regenerative pathways.

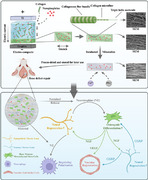

## 1 Introduction

Bone, as the body's primary hard tissue, serves critical roles in structural support and protection. Bone injuries are one of the most common organ injuries.^[^ ^1^ ^]^ Current regenerative strategies emphasize osteogenic cell recruitment and biomineralization, while accumulating evidence highlights the indispensable role of the peripheral nervous system in orchestrating effective bone healing.^[^ ^2^ ^]^ Sensory and sympathetic nerve fibers are extensively distributed in bone tissue, where they influence skeletal development, metabolism, and repair by releasing neuropeptides and neurotransmitters that act on bone‐lineage cells and the surrounding microenvironment.^[^ ^3^ ^]^ Specifically, peptidergic nerves have a direct impact on bone formation through the release of neurogenic factors.^[^ ^3^ ^]^ Key neuropeptides, including substance P, calcitonin gene‐related peptide (CGRP), neuropeptide Y, and vasoactive intestinal peptide, promote bone formation and regulate metabolism by binding to specific receptors on bone cells and modulating their functions. Notably, CGRP, one of the most crucial neuropeptides secreted by peptidergic sensory neurons, not only facilitates osteogenesis via cAMP/PKA and Wnt/β‐catenin pathways, but also acts as a potent vasodilator, coupling sensory nerve activity to angiogenesis.^[^ ^4^, ^5^ ^]^ This neurovascular coupling is critical during the early phase of bone injury repair, where sensory nerve sprouting precedes both neovascularization and new bone formation, creating a hyperinnervated microenvironment that is essential for initiating regeneration.^[^ ^6^, ^7^ ^]^ These findings underscore the indispensable role of neural regulation in orchestrating successful bone repair.

Despite significant advancements in bone tissue engineering, conventional materials often fail to account for the neural components necessary for integrated repair.^[^ ^2^, ^8^ ^]^ Traditional scaffolds focus predominantly on bone‐related properties such as mechanical strength and osteogenic support.^[^ ^9^ ^]^ However, their limited ability to guide neural growth and stimulate nerve‐related factors has hindered their effectiveness in complex injury scenarios where neural regeneration is essential. Addressing this gap requires a dual‐functional approach that combines bone and nerve regeneration capabilities.

Emerging evidence suggests that norepinephrine (NE), a key neurotransmitter released by sympathetic nerve terminals, may play a crucial role in orchestrating the interaction between nerves and bone tissues during repair.^[^ ^10^, ^11^ ^]^ Recent studies have demonstrated that NE and nerve growth factor (NGF) can synergistically regulate the interplay between nerves and cancer‐associated fibroblasts in colorectal cancer, influencing disease progression.^[^ ^10^ ^]^ In another context, NE released from sympathetic terminals modulates the expression of glial cell line‐derived neurotrophic factor in neighboring gonadal adipose mesenchymal cells via activation of β2‐adrenergic receptors.^[^ ^11^ ^]^ These findings imply that NE may serve as a key modulatory signal influencing interactions between neural and stromal cells in various tissue environments. We therefore hypothesize that during bone injury repair, NE may mediate crosstalk between peripheral nerves and bone marrow mesenchymal stem cells (BMSCs), in part by regulating the expression of neurotrophic factors secreted by BMSCs. Furthermore, appropriate concentrations of NE have been shown to promote the proliferation of rat bone marrow mesenchymal stem cells (rBMSCs).^[^ ^12^ ^]^ NE has also demonstrated potential in stimulating bone regeneration and modulating macrophage polarization, supporting both skeletal repair and immune regulation.^[^ ^13^, ^14^ ^]^ A previous study employed oxidative polymerization to convert NE into polyNE, which was then co‐deposited with CaCO_3_ through electrospinning to fabricate mineralized nanofibrous collagen scaffolds. These scaffolds were shown to promote adhesion and osteogenesis of human fetal osteoblasts.^[^ ^15^ ^]^ Together, these findings highlight NE as a promising bioactive factor that may support both bone regeneration and nerve repair.

In mammals, bones and dentin are composed of hydroxyapatite crystals grown along the direction of collagen fibers, with a hierarchical and ordered structure that ensures their toughness and strength.^[^ ^16^ ^]^ Inspired by this natural character, biomimetic mineralization strategies have been developed to replicate the composition and organization of native bone. Among these, biomimetic aligned mineralized collagen scaffolds have gained particular attention due to their resemblance to natural bone in both structure and composition, excellent biocompatibility, and appropriate mechanical properties for supporting regeneration.^[^ ^17^, ^18^ ^]^ Furthermore, aligned collagen fibers provide not only structural support but also serve as topographical cues that guide cell orientation and axonal extension, thereby contributing to both osteogenic and neurogenic responses.^[^ ^19^, ^20^, ^21^ ^]^ The direct application of NE in vivo is challenged by its rapid diffusion and metabolic clearance, leading to short‐lived effects and subtherapeutic local concentrations. This not only diminishes its therapeutic efficacy but also increases the risk of systemic side effects. To overcome these limitations, we incorporated NE into biomimetic aligned mineralized collagen scaffolds. The scaffold serves as a reservoir, enabling the gradual and sustained release of NE, which ensures a stable and localized concentration in the targeted area. By combining structural biomimicry with controlled delivery of neuroactive signals, the resulting scaffold is designed to synchronously stimulate bone regeneration and nerve repair.

**Figure 1:**
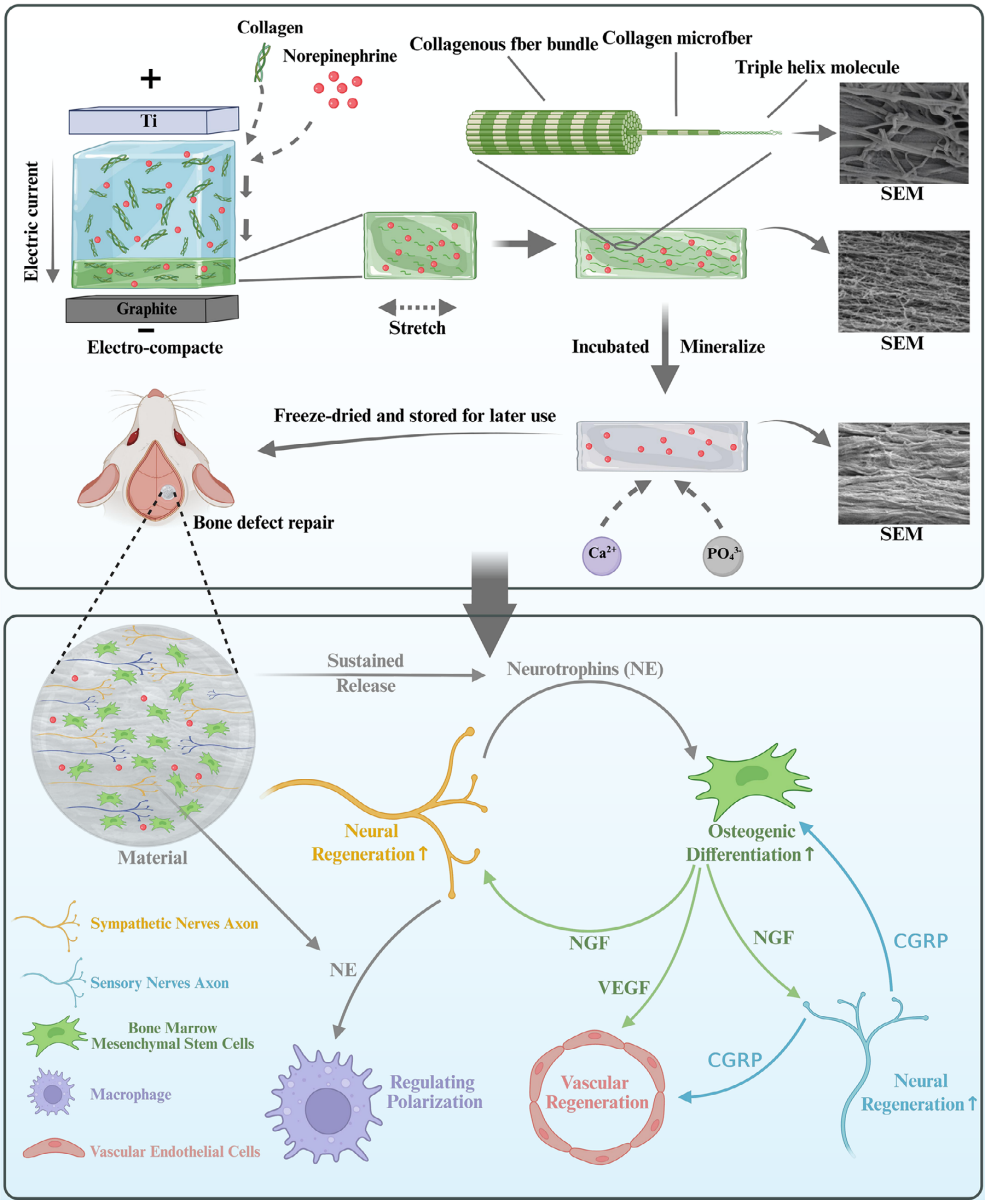
Schematic illustration of the fabrication process and regenerative mechanisms of NE‐MEC. The NE‐containing collagen solution was electrocompacted via isoelectric focusing between titanium and graphite electrodes to form a densely aligned collagen construct. After uniaxial stretching (150% elongation) to further enhance fibrillar orientation, the constructs were mineralized in polyacrylic acid‐stabilized amorphous calcium phosphate solution to mimic native bone composition. The resulting scaffolds were freeze‐dried and stored for subsequent implantation. SEM images show the microstructural morphology before and after mineralization. Notably, even in the absence of NE, electrocompacted mineralized collagen scaffolds provide topographical guidance for axonal alignment and rBMSC orientation, and contribute to osteogenic differentiation. The NE‐MEC scaffold enables sustained release of NE, which enhances rBMSC osteogenesis and upregulates nerve growth factor expression during early stages. NGF promotes peripheral nerve regeneration and induces CGRP production from sensory neurons. In turn, CGRP facilitates osteogenic differentiation and neovascularization. Meanwhile, NE‐MEC stimulates vascular endothelial growth factor A expression in the regenerating bone tissue—partly from BMSC—and modulates macrophage polarization. Together, these effects establish a regenerative circuit that orchestrates osteogenic, neurogenic, angiogenic, and immunomodulatory processes to promote effective bone defect repair. Created with biorender.com.

As illustrated in **Figure** **1**, we developed a biomimetic mineralized collagen scaffold inspired by the hierarchical structure of native bone tissue. Type I collagen served as the structural matrix, into which NE was incorporated prior to isoelectric focusing to fabricate a highly aligned collagen membrane. The resulting collagen membrane was then subjected to 150% uniaxial stretching to enhance collagen fiber alignment, followed by mineralization in a polyacrylic acid (PAA)‐stabilized amorphous calcium phosphate (ACP) solution. After freeze‐drying, a structurally aligned, NE‐loaded mineralized collagen scaffold (NE‐MEC) was obtained. This scaffold mimics both the composition and microstructural organization of native bone, providing topographical guidance for axonal growth and rBMSCs alignment. Beyond its osteogenic potential, NE‐MEC also promotes neural regeneration, angiogenesis, and immunomodulation through the bioactivity of NE and cell–cell interactions. These multifunctional effects were validated through transcriptomic analysis, material characterization, in vitro functional assays, and a rat calvarial defect model. In summary, we designed a biomimetic mineralized collagen scaffold incorporating NE, which enables simultaneous modulation of osteogenesis, neurogenesis, angiogenesis, and immune polarization, offering a multifunctional platform for enhanced skeletal tissue regeneration.

## 2 Results

### 2.1 Norepinephrine Modulates rBMSC Potential

**Figure 2:**
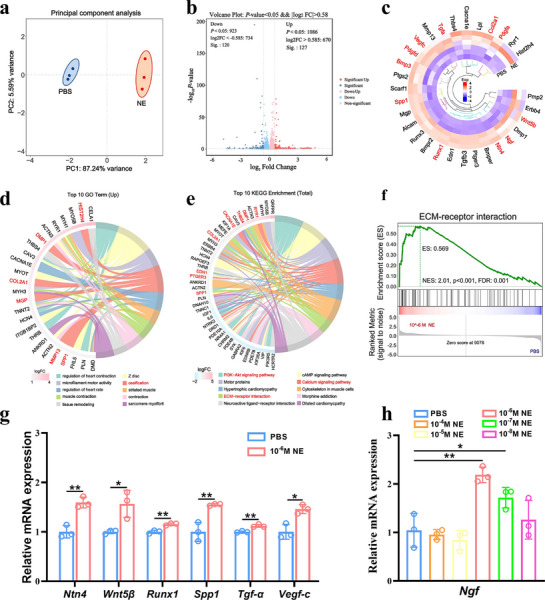
Transcriptomic analysis and validation of NE‐induced gene expression in rBMSCs. a) Principal component analysis showing distinct transcriptomic profiles between PBS‐ and 10^−6^ M NE‐treated rBMSCs. b) Volcano plot of differentially expressed genes (DEGs). c) Heatmap showing representative DEGs associated with neurogenic, angiogenic, and osteogenic processes. d) Top 10 upregulated GO enriched in the NE group. e) Top 10 enriched KEGG pathways among DEGs. f) GSEA showing upregulation of the ECM–receptor interaction pathway in NE‐treated cells. g) qPCR validation of selected DEGs. h) *Ngf* mRNA expression in rBMSCs after 24 h NE treatment at various concentrations. *P < 0.05; **P < 0.01. Data are mean ± SD (*n* = 3).

To explore the regulatory potential of NE in bone repair, we first examined its effects on the transcriptomic profile of rBMSCs following treatment with 10^−6^ m NE for 24 h. Principal component analysis (PCA) demonstrated a distinct separation between the NE‐treated and control (PBS) groups, suggesting significant changes in gene expression profiles (**Figure** **2**a). The volcano plot further revealed over 200 significantly changed genes (Figure 2b). Notably, heatmap analysis highlighted the upregulation of key genes involved in osteogenic differentiation (e.g., *Runx1*, *Spp1*, *Col2a1*), neurotrophic factors (e.g., *Ngf*, *Ntn4*), and angiogenesis (e.g., *Vegfc*) (Figure 2c). Gene Ontology (GO) enrichment analysis showed significant associations with processes such as ossification (Figure 2d), while Kyoto Encyclopedia of Genes and Genomes (KEGG) pathway analysis emphasized pathways including PI3K‐Akt signaling pathway, ECM–receptor interaction, and Calcium signaling pathway (Figure 2e). Gene set enrichment analysis (GSEA) further confirmed the activation of the ECM–receptor interaction pathway in the NE group (Figure 2f). To validate transcriptomic findings, we performed qPCR for selected differentially expressed genes. NE significantly upregulated the expression of *Ntn4*, *Wnt5β*, *Runx1*, *Spp1*, *Tgf‐α*, and *Vegfc* compared to PBS (Figure 2g). Importantly, NE also dose‐dependently increased *Ngf* expression, with 10^−6^ m showing the most pronounced effect (Figure 2h), suggesting its capacity to enhance the neurotrophic function of rBMSCs. These results indicate that NE not only promotes the osteogenic differentiation of rBMSCs but also primes them for neurotrophic support by upregulating NGF.

### 2.2 Biomimetic Design and Characterization of NE‐MEC Scaffolds

**Figure 3:**
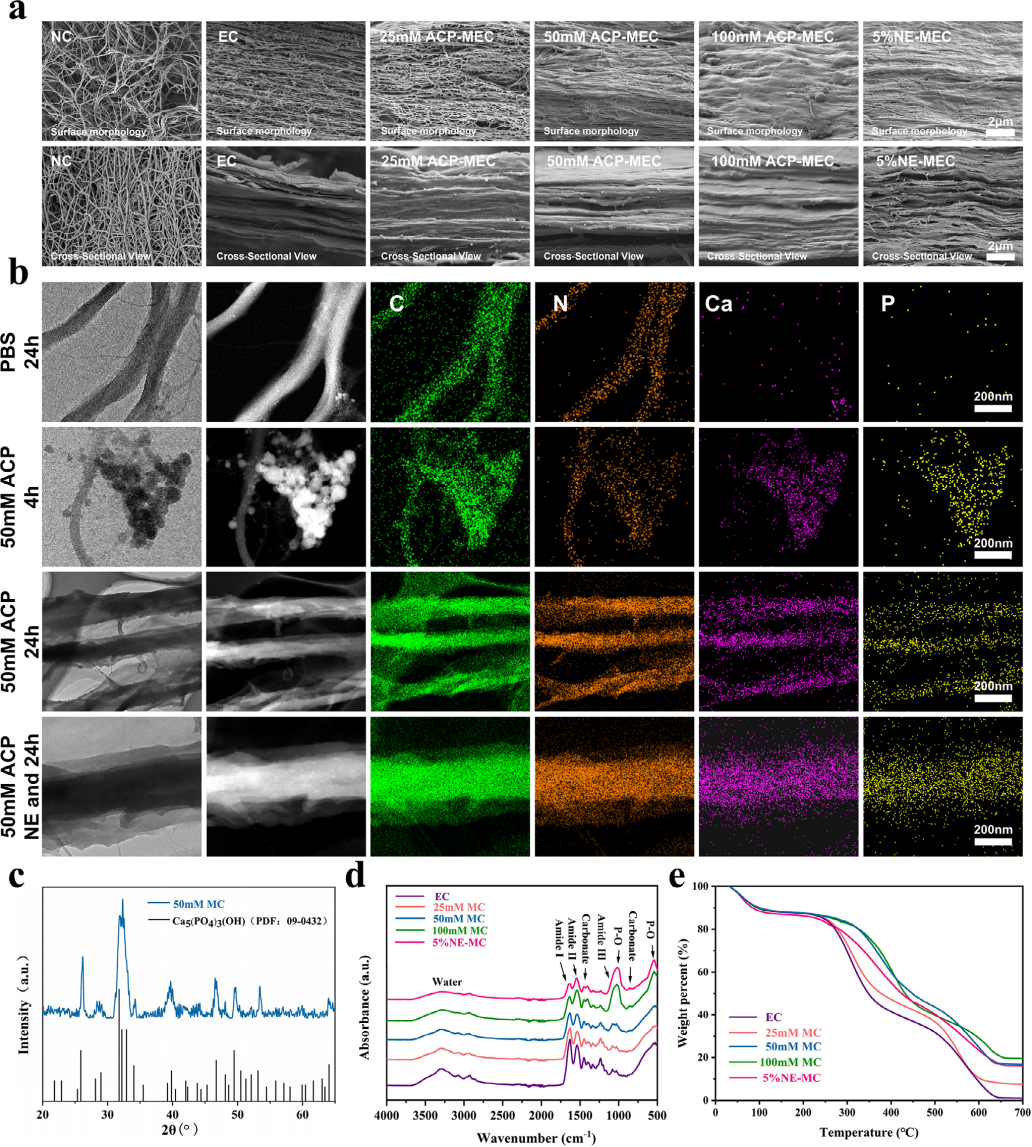
Morphological and compositional characterization of scaffolds. a) SEM images showing surface and cross sectional morphologies of scaffolds. b) TEM and elemental mapping (C, N, Ca, P) of collagen scaffolds mineralized with 50 mm ACP for 4 h and 24 h. **c** XRD pattern of 50 mm ACP‐MEC. d) FTIR spectra of various scaffolds. e) TGA curves demonstrating mineral content of different scaffolds. Scale bars: 2 µm in (a), 200 nm in (b).

To construct a multifunctional scaffold that mimics the biochemical composition and anisotropic architecture of native bone, we fabricated NE‐loaded mineralized electrocompacted collagen (NE‐MEC) scaffolds using isoelectric focusing, mechanical stretching, and PAA‐stabilized ACP mineralization. SEM analysis revealed that compared to randomly organized native collagen (NC), electrocompacted collagen (EC) exhibited a highly aligned fibrillar architecture (**Figure** **3**a). Upon mineralization with increasing concentrations of ACP, distinct differences in mineral deposition were observed: 25 mm ACP‐MEC showed incomplete mineralization with exposed collagen fibrils and visible porosity; 50 mm ACP‐MEC achieved uniform mineral deposition while preserving the anisotropic structure; in contrast, 100 mm ACP‐MEC underwent excessive mineralization that masked the underlying fibrillar alignment. These findings suggest that 50 mm ACP represents an optimal condition for achieving structural biomimicry. 5%NE‐MEC scaffolds mineralized under 50 mm ACP conditions also preserved the anisotropic structure, indicating that the incorporation of NE does not disrupt collagen alignment (Figure 3a). High‐magnification SEM images further revealed the preservation of characteristic collagen D‐periodicity in EC and 50 mM ACP‐MEC (Figure S2, Supporting Information), indicating that neither electrocompaction nor mineralization disrupted the native architecture of collagen. To further verify mineral localization, TEM analysis and elemental mapping were performed on scaffolds incubated in 50 mm ACP solution for 4 h and 24 h. At 4 h, Ca and P signals were primarily concentrated in clusters deposited on the surface of collagen fibrils and exhibited non‐uniform distribution. Upon increasing time to 24 h, these elements became homogeneously distributed along the entire fibril length, confirming successful intrafibrillar mineralization (Figure 3b). In addition, EDS mapping was also performed on mineralized scaffolds with and without NE incorporation, and no appreciable difference in Ca and P distribution was observed between the two groups, suggesting that NE loading does not impair the mineral deposition process (Figure 3b). Quantitative EDS results further supported successful intrafibrillar mineralization under this condition (Figure S3, Supporting Information). Thus, 50 mm ACP for 24 h was selected as the optimized mineralization condition for the subsequent scaffold fabrication.

XRD analysis of 50 mm ACP‐MEC showed diffraction peaks characteristic of low‐crystallinity hydroxyapatite (HA), confirming the successful deposition of a bone‐mimetic mineral phase (Figure 3c). FTIR revealed characteristic amide I and II peaks, confirming collagen presence (Figure 3d). As mineralization increased, phosphate (P–O) peaks became more prominent, indicating progressive HA incorporation and the formation of biphasic collagen–HA composites. TGA showed increasing mineral content with higher ACP concentrations, and NE incorporation did not alter HA deposition efficiency (Figure 3e).

**Figure 4:**
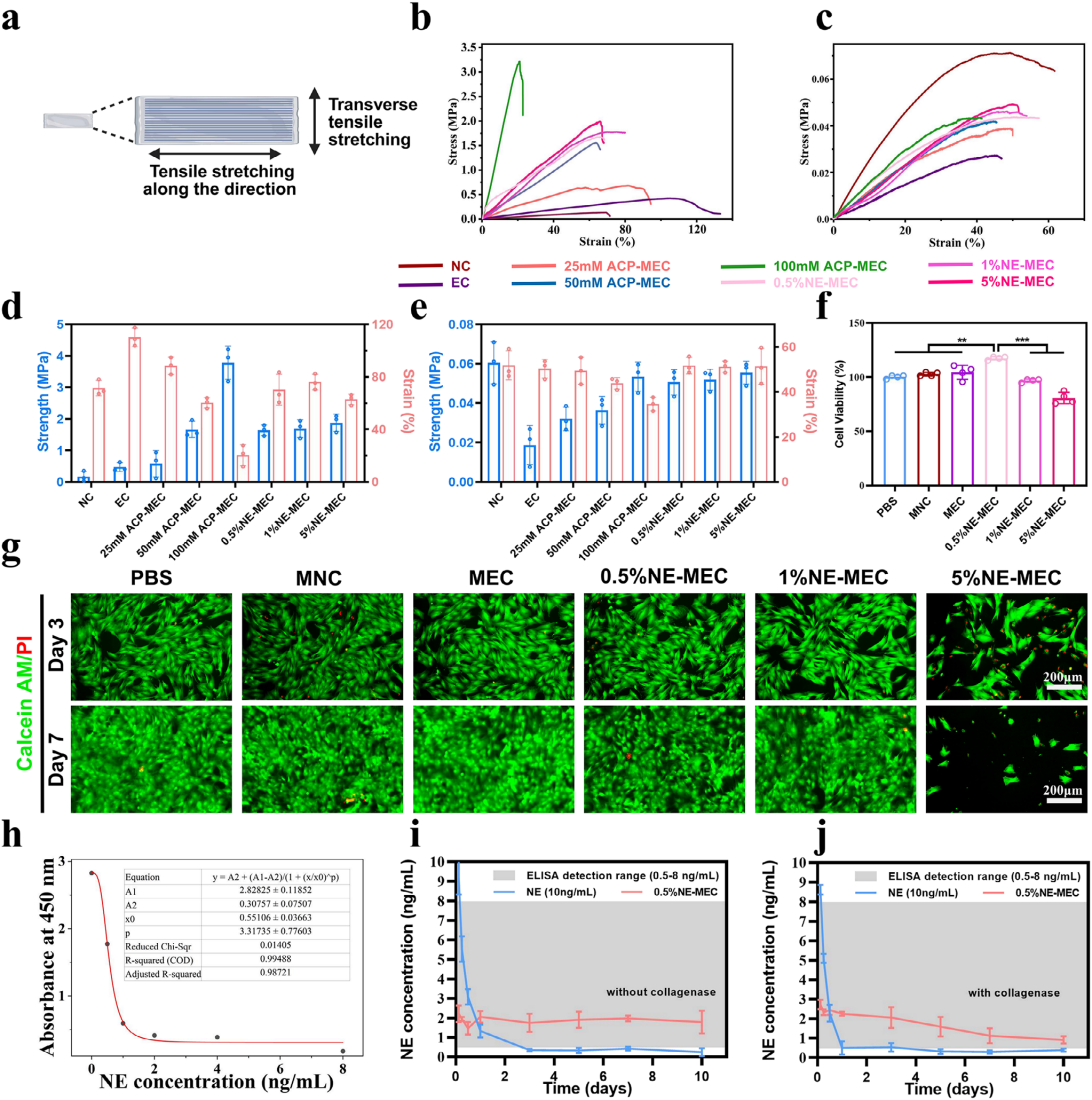
Mechanical properties, swelling stability, and cytocompatibility of NE‐MEC scaffolds. a) Schematic of tensile testing setup. b) Longitudinal tensile stress–strain curves of scaffolds. c Transverse tensile stress–strain curves of scaffolds. The average ultimate tensile strength and strain at break of various scaffold groups (*n* = 3), measured in both longitudinal d) and transverse directions e). CCK‐8 assay f) and live/dead fluorescence staining g) of rBMSCs cultured with different scaffolds for 3 and 7 days. h) ELISA standard curve fitted with a four‐parameter logistic model. i) Release of free NE (10 ng mL^−1^) and 0.5% NE‐MEC in PBS without collagenase. j) Release in PBS with collagenase to mimic enzymatic degradation. Scale bars: 100 µm. **P < 0.01; ***P < 0.001. a Created with biorender.com. Data are mean ± SD (*n* = 3).

We next evaluated the mechanical, swelling, and cytocompatibility properties of NE‐MEC scaffolds. To evaluate mechanical properties, tensile testing was performed on longitudinally and transversely stretched scaffolds, respectively (**Figure** **4**a). As shown in the longitudinal tensile curves (Figure 4b), EC exhibited markedly improved tensile strength compared to NC, confirming the reinforcement effect of fiber alignment. Upon mineralization, the mechanical strength of the scaffolds was further enhanced with rising ACP concentration. The 100 mm ACP‐MEC reached the highest ultimate tensile strength, but compromise with a significantly reduced elongation at break. The introduction of NE led to a partial recovery of elasticity for the mineralized ACP‐MEC, possibly due to the disruption of intermolecular interactions, leading to a more loosely packed interlayer structure or molecular domains. This enabled multi‐scale stress dissipation to resist tensile deformation, thereby enhancing the elasticity. In transverse tensile testing (Figure 4c), all mineralized groups displayed lower mechanical performance compared to their longitudinal counterparts, highlighting the anisotropic nature of the scaffolds. Quantitative bar graphs of the average tensile strength and toughness (Figure 4d,e) supported these trends. Swelling analysis further demonstrated that EC, MEC, and NE‐MEC scaffolds retained stable dimensions with limited weight gain over 7 days in PBS, whereas NC scaffolds exhibited continuous water absorption and swelling during the same period (Figure S4, Supporting Information). By day 7, the NC structure was visibly collapsed, with dispersed collagen fragments and structural disintegration, rendering it unsuitable for further evaluation at day 9. CCK‐8 assay confirmed optimal viability in the 0.5% NE‐MEC group (Figure 4f). Correspondingly, live/dead staining showed high viability of rBMSCs co‐culture with all scaffolds except 5%NE‐MEC, which presented cytotoxicity (Figure 4g). Due to the observed cytotoxicity of the 5% NE‐MEC group, this condition was excluded from the subsequent experiments. These findings confirm that NE‐MEC scaffolds maintain favorable biomechanical strength and cell compatibility.

In addition, the sustained release of NE from NE‐MEC scaffolds was evaluated by ELISA, with the standard curve shown in Figure 4h. Free NE solution showed a rapid decline, falling below the detection limit within 1–3 days due to its inherent instability. In contrast, NE‐MEC scaffolds maintained detectable and relatively stable NE concentrations over a period of 10 days, indicating continuous release from the scaffold matrix. In the presence of collagenase, NE concentrations from NE‐MEC scaffolds decreased, but remained above the detection limit (Figure 4i,j). These results confirm the sustained release capability of NE‐MEC scaffolds.

### 2.3 NE‐MEC Scaffolds Enhance Osteogenic Differentiation

**Figure 5:**
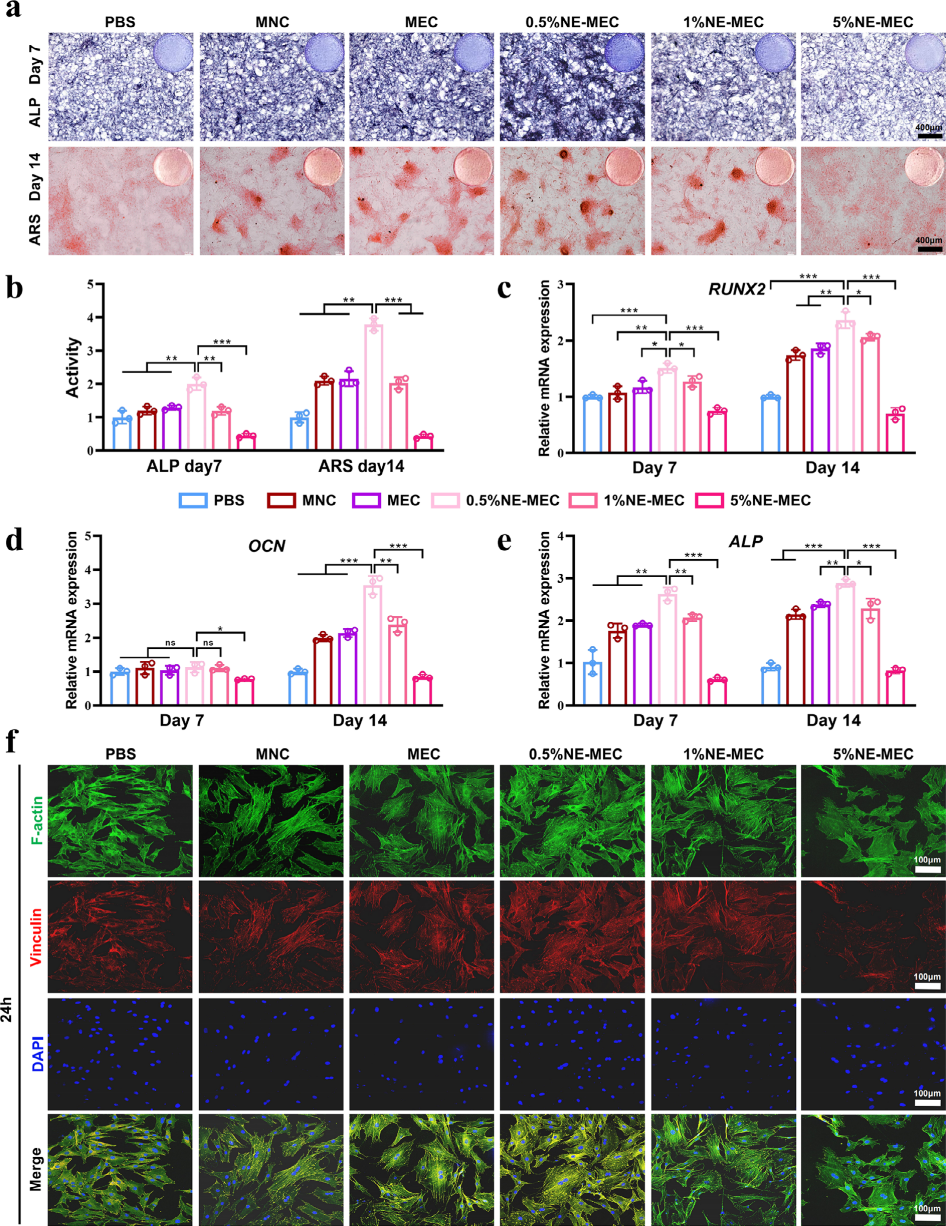
Osteogenic differentiation of rBMSCs co‐cultured with different scaffolds. a) Representative image of ALP staining on day 7 and ARS staining on day 14. b) Quantification of ALP activity and ARS mineralization. c–e) Relative mRNA expression levels of osteogenic markers *RUNX2*, *OCN*, and *ALP* at days 7 and 14 as measured by qPCR. f) Immunofluorescence staining of rBMSCs after 24 h of co‐culture, showing cytoskeletal F‐actin (green), focal adhesion marker vinculin (red), and nuclei (DAPI, blue). Scale bars: 300 µm in (a), 100 µm in (f). *P < 0.05; **P < 0.01; ***P < 0.001. Data are mean ± SD (*n* = 3).

To investigate the osteoinductive potential of NE‐MEC scaffolds, rBMSCs were co‐cultured with various scaffolds under osteogenic induction conditions. ALP staining on day 7 showed enhanced early osteogenic activity in the MNC, MEC, 0.5%NE‐MEC and 1%NE‐MEC groups, with the strongest effect observed in 0.5%NE‐MEC (**Figure** **5**a,b). Similarly, ARS staining on day 14 revealed robust calcium nodule formation in the MNC, MEC, 0.5% and 1% NE‐MEC groups, with the strongest effect observed in 0.5%NE‐MEC (Figure 5a,b). In contrast, 5%NE‐MEC resulted in notably weaker staining, indicating a dose‐dependent threshold beyond which NE may hinder osteogenesis. Quantitative PCR analysis of osteogenic marker genes further confirmed these trends. Compared to the PBS group, the expression levels of *RUNX2*, *OCN*, and *ALP* were significantly upregulated on day 14 in the MNC, MEC, 0.5%NE‐MEC, and 1%NE‐MEC groups, with the 0.5%NE‐MEC group showing the most pronounced effect (Figure 5c–e). These findings suggest that MNC and MEC scaffolds alone can promote osteogenic differentiation, while the addition of low‐dose NE—especially at 0.5%—further enhances this effect. In contrast, the 5%NE‐MEC group exhibited suppressed expression of all markers at both time points, indicating a dose‐dependent inhibitory effect at higher NE concentrations. After 24 h of co‐culture, immunofluorescence staining of rBMSCs revealed more extensive actin filament spreading and richer focal adhesion formation in the MNC, MEC, 0.5%NE‐MEC, and 1%NE‐MEC groups compared to the PBS control, with the 0.5%NE‐MEC group showing the most pronounced enhancement (Figure 5f). In contrast, cells cultured on 5%NE‐MEC scaffolds appeared sparse and morphologically abnormal, with fewer focal adhesions, consistent with impaired cell viability. Figure S5 (Supporting Information) further demonstrated that, compared to MNC, rBMSCs cultured on MEC and 0.5%NE‐MEC scaffolds exhibited stronger directional alignment along the scaffold architecture, indicating enhanced topographical guidance. Together, these findings confirm that MNC and MEC scaffolds inherently promote osteogenic differentiation, and that incorporation of an appropriate NE dose can further enhance this effect.

To further elucidate the receptor‐specific effects of NE on osteogenic differentiation, we conducted ALP and ARS staining across groups treated with NE‐MEC scaffolds in the presence or absence of adrenergic receptor antagonists. As shown in Figure S6 (Supporting Information), 0.5% NE‐MEC significantly enhanced ALP activity and calcium deposition compared to the control and MEC groups. This pro‐osteogenic effect was notably attenuated upon treatment with the α1‐AR antagonist Prazosin, suggesting the involvement of α1‐adrenergic signaling. In contrast, 5% NE‐MEC exhibited reduced osteogenic potential, which was partially reversed by β‐AR blockade using Propranolol, further supporting the concentration‐dependent, receptor‐specific dual role of NE in osteogenesis.

### 2.4 NE‐MEC Supports Neurotrophic Signaling and Axonal Alignment

**Figure 6:**
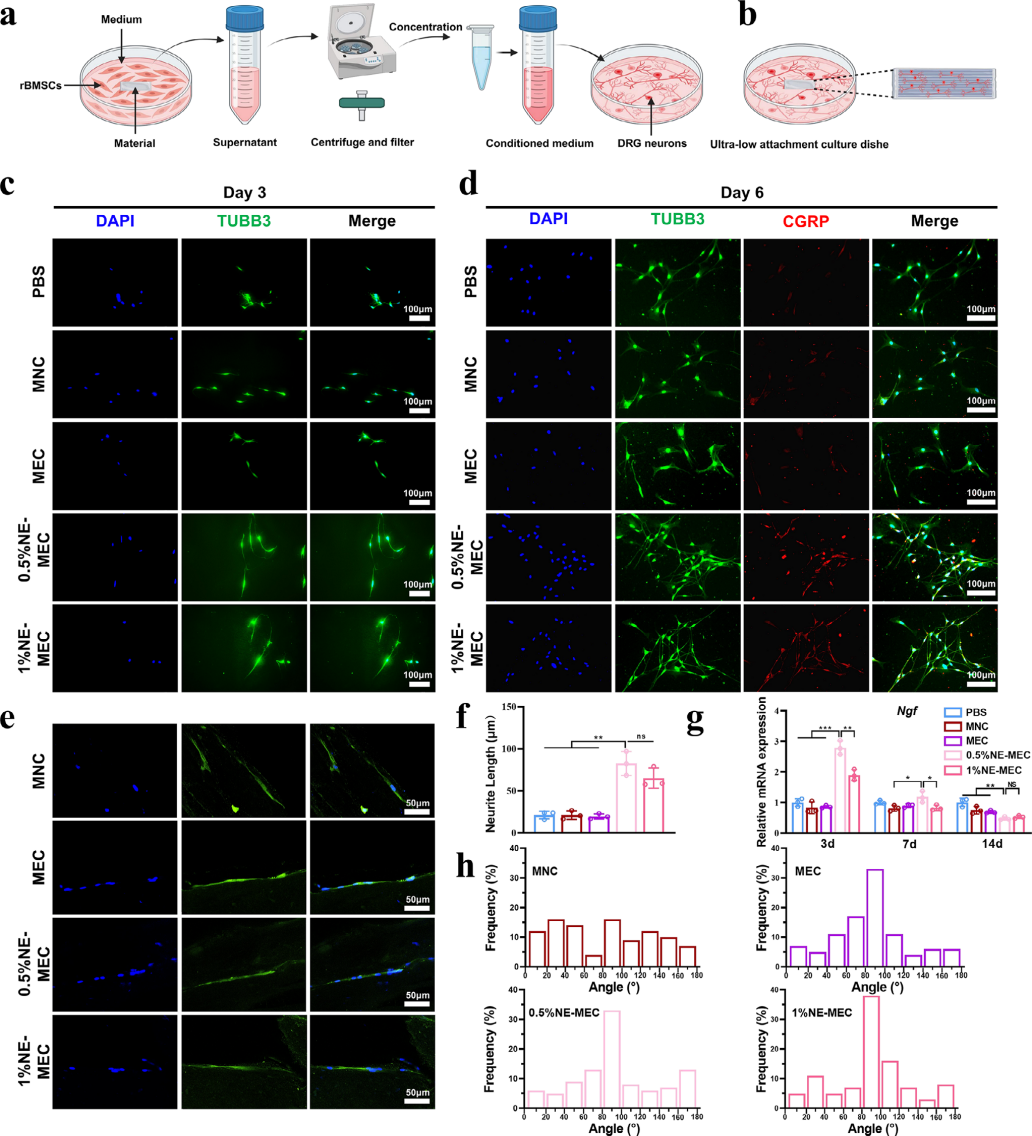
Scaffold‐mediated neurogenic support and axonal guidance. Schematic illustration of the indirect a) and direct b) co‐culture strategies used to evaluate scaffold effects on DRG neurons. c,d) Immunofluorescence analysis of DRG neurons cultured with conditioned media. (c) TUBB3 staining at day 3. (d) TUBB3 and CGRP staining at day 6. e) TUBB3 staining of DRG neurons directly cultured on scaffold surfaces for 4 days. f) Quantification of neurite length from the indirect co‐culture model at day 3. g) qPCR analysis of *Ngf* expression in rBMSCs cultured with scaffolds for 3, 7, and 14 days. h) Neurite orientation analysis derived from angular measurements in panel E. Scale bars: 100 µm in (c and d), 50 µm in (e). *P < 0.05; **P < 0.01; ***P < 0.001; ns, not significant. a and b Created with biorender.com. Data are mean ± SD (*n* = 3).

To investigate NE‐MEC–mediated neurogenic support and axonal guidance, we employed both indirect and direct co‐culture strategies (schematically illustrated in **Figure** **6**a,b). In the indirect co‐culture model, TUBB3 immunofluorescence staining on day 3 revealed that conditioned media from 0.5% and 1% NE‐MEC scaffolds significantly promoted neurite extension in DRG neurons compared to PBS, MNC, and MEC groups (Figure 6c,f). A similar pro‐neurogenic effect was observed in PC12 cells—a sympathetic neuronal model—cultured with the same conditioned media (Figure S7, Supporting Information), further supporting the neurotrophic potential of NE‐MEC. By day 6, co‐staining for TUBB3 and CGRP showed more extensive neurite networks and enhanced CGRP expression in the NE‐containing groups, particularly in the 0.5%NE‐MEC condition (Figure 6d). Consistently, ELISA results confirmed increased CGRP secretion in the NE‐MEC groups (Figure S8, Supporting Information). To explore the underlying mechanism, qPCR analysis of rBMSCs cultured with different scaffolds revealed a time‐dependent upregulation of NGF expression in the 0.5% and 1% NE‐MEC groups, with the highest levels detected on day 3 (Figure 6g), suggesting that the enhanced neural response may be driven by NE‐MEC–stimulated neurotrophic factor secretion from rBMSCs. In the direct co‐culture setup, DRG neurons were seeded directly onto scaffold surfaces. After 4 days, TUBB3 staining showed that MEC and NE‐MEC scaffolds promoted axonal alignment along the anisotropic collagen fibers (Figure 6e). Angular analysis of neurite orientation further confirmed that neurites on MEC and NE‐MEC scaffolds displayed enhanced directional alignment compared to those on MNC scaffolds (Figure 6h). Collectively, these findings indicate that NE‐MEC scaffolds facilitate both neurotrophic factor secretion from rBMSCs and topographical guidance of neuronal growth, thereby establishing a neuro‐supportive microenvironment through paracrine and structural mechanisms.

### 2.5 NE‐MEC Tunes Macrophages, not Vessels In Vitro

**Figure 7:**
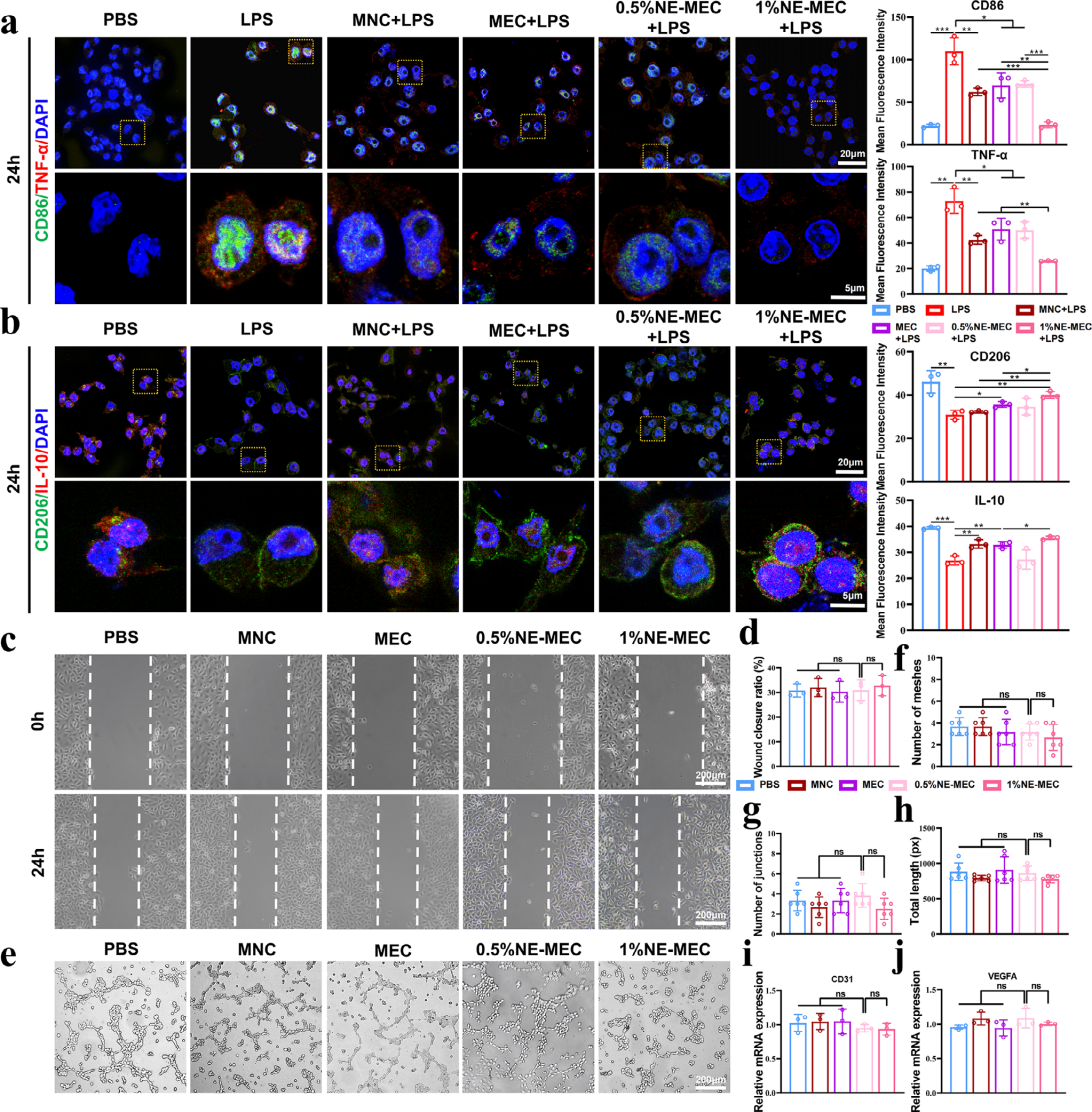
Immunomodulatory and pro‐angiogenic evaluation of NE‐MEC scaffolds. a,b) Immunofluorescence staining of RAW264.7 macrophages cultured with scaffolds under LPS stimulation (24 h) to assess polarization states. Enlarged regions highlight representative cells. Quantitative analysis of fluorescence intensity is shown at right. c) Representative images from wound healing assays of HUVECs cultured in scaffold‐conditioned serum‐free medium at 0 h and 24 h. d) Tube formation assay of HUVECs, with visualization of capillary‐like network structures after 6 h. e) Wound closure ratio at 24 h. Tube formation parameters including mesh number (f), number of junctions (g), and total network length (h). qPCR analysis of angiogenesis‐related genes *CD31* (i) and *VEGFA* (j) in HUVECs cultured under different conditions. Scale bars: 20 and 5 µm in (a and b). *P < 0.05; **P < 0.01; ***P < 0.001; ns, not significant. Data are mean ± SD (*n* = 3).

To investigate the immunomodulatory properties of NE‐MEC scaffolds, RAW264.7 macrophages were cultured under LPS stimulation in the presence of different scaffolds. While the in vivo cranial defect model is not overtly infected, injury‐induced bone healing still involves an early pro‐inflammatory phase. Thus, the LPS model serves as a standardized proxy to examine the immunomodulatory properties of the scaffolds under conditions relevant to early‐stage inflammation. After 24 h, immunofluorescence staining showed that NE‐MEC, especially the 1%NE‐MEC groups, significantly suppressed M1 polarization markers CD86 and TNF‐α while enhancing the expression of M2 markers CD206 and IL‐10 (**Figure** **7**a,b). Consistent with these findings, ELISA analysis further confirmed that NE‐MEC scaffolds decreased TNF‐α and increased IL‐10 secretion in the culture supernatants (Figure S9, Supporting Information). These findings indicate that NE‐MEC scaffolds effectively promote macrophage polarization toward a pro‐regenerative M2 phenotype. Next, we assessed the angiogenic potential of NE‐MEC scaffolds using in vitro co‐culture with HUVECs. In wound healing assays, none of the scaffold‐conditioned groups showed significant enhancement in endothelial cell migration compared to the PBS control (Figure 7c,d). Similarly, tube formation assays revealed no notable differences among groups in mesh number, junction count, or total network length (Figure 7e–h). Moreover, qPCR analysis of angiogenic markers CD31 and VEGFA in HUVECs revealed no significant upregulation (Figure 7i,j). Collectively, these findings suggest that NE‐MEC scaffolds effectively promote macrophage polarization toward an anti‐inflammatory M2 phenotype, but exhibit limited direct angiogenic induction in HUVECs in vitro. Nonetheless, significant pro‐angiogenic effects observed in subsequent in vivo studies imply that the vascularization potential of NE‐MEC scaffolds likely relies on the presence of complex multicellular interactions within the native tissue microenvironment.

### 2.6 Mechanistic Insights into NE‐MEC Induced Osteogenesis

**Figure 8:**
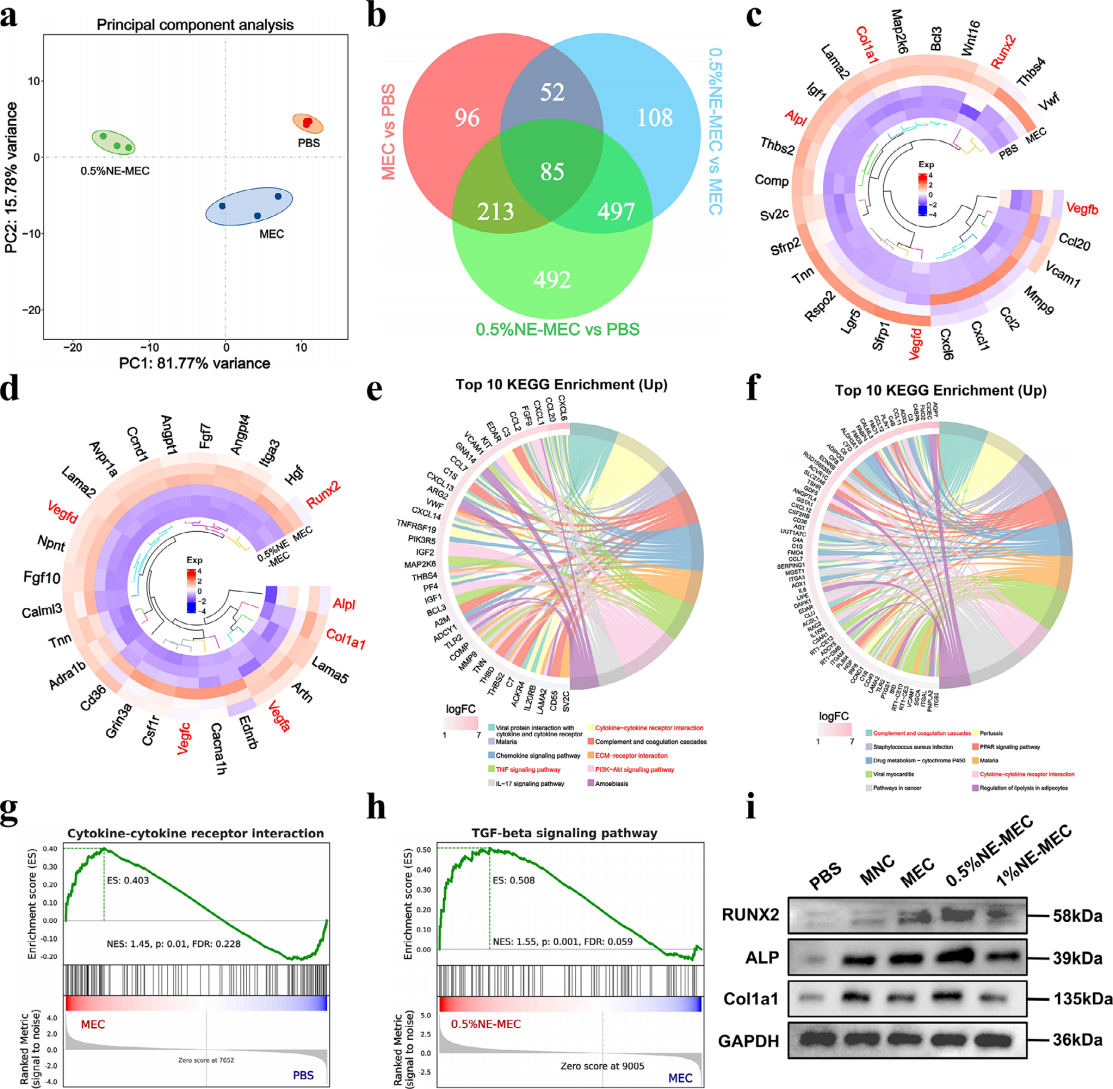
Transcriptomic analysis of rBMSCs co‐cultured with scaffolds. a) PCA illustrating transcriptomic variance among PBS, MEC, and 0.5%NE‐MEC groups. b) Venn diagram showing the number of DEGs between each pairwise comparison. Heatmaps depicting representative osteogenesis‐ and angiogenesis‐related gene expression profiles from MEC versus PBS (c) and 0.5%NE‐MEC versus MEC d) comparisons. KEGG enrichment analysis showing the top 10 upregulated pathways in MEC versus PBS e) and 0.5%NE‐MEC versus MEC f) groups. GSEA of key pathways enriched in MEC versus PBS g, cytokine–cytokine receptor interaction) and 0.5%NE‐MEC versus MEC h, TGF‐β signaling pathway). i) Western blot validation of osteogenic markers (RUNX2, ALP, and Col1a1) in rBMSCs co‐cultured with various scaffolds, with GAPDH as the internal control.

To elucidate the molecular mechanisms underlying the osteoinductive effects of NE‐MEC scaffolds, we performed transcriptomic profiling of rBMSCs co‐cultured with PBS, MEC, or 0.5%NE‐MEC. PCA revealed distinct clustering patterns, with the 0.5%NE‐MEC group showing the greatest transcriptional deviation from PBS (**Figure** **8**a). Venn analysis revealed distinct and overlapping DEGs among the MEC, 0.5%NE‐MEC, and PBS groups, indicating transcriptional distinctions among all three conditions (Figure 8b). This, together with PCA clustering, reflects the unique gene expression profiles induced by mineralized collagen and further modulated by NE incorporation. Heatmap analysis showed that MEC scaffolds significantly upregulated key osteogenesis‐related genes including *Runx2*, *Col1a1*, and *Alpl* when compared to PBS (Figure 8c). Further elevation of osteogenic markers (Runx2, Col1a1, and Alpl) and several angiogenesis‐associated genes (*Vegfa*, *Vegfc*, and *Vegfd*) was observed in the 0.5%NE‐MEC group compared to MEC (Figure 8d). KEGG enrichment analysis identified the top 10 significantly upregulated signaling pathways in each comparison group. In the MEC versus PBS comparison (Figure 8e), the highlighted upregulated pathways included cytokine–cytokine receptor interaction, PI3K‐Akt signaling pathway, TNF signaling pathway, and ECM–receptor interaction, all of which are closely associated with osteogenic signaling and stem cell differentiation. In the 0.5%NE‐MEC versus MEC comparison (Figure 8f), NE incorporation further enriched pathways such as the cytokine–cytokine receptor interaction and Complement and coagulation cascades, indicating additional osteogenic regulatory effects imparted by NE. GSEA confirmed that cytokine–cytokine receptor interaction was significantly enriched in the MEC group compared to PBS (Figure 8g). Meanwhile, TGF‐β signaling pathway was significantly upregulated in the 0.5%NE‐MEC group relative to MEC (Figure 8h), consistent with the known role of TGF‐β in bone formation and remodeling. Western blot analysis further validated the upregulation of RUNX2, ALP, and Col1a1 in the 0.5%NE‐MEC group, consistent with transcriptomic results (Figure 8i). Together, these data confirm that both MEC and NE‐MEC scaffolds activate osteogenic transcriptional programs in rBMSCs, with NE incorporation further enhancing this response. Additionally, increased expression of vascular‐related genes such as *Vegfa* and *Vegfc* suggests that 0.5%NE‐MEC scaffolds may also support angiogenic signaling indirectly through rBMSC activation.

### 2.7 NE‐MEC Scaffolds Promote Neurovascularized Bone Regeneration In Vivo

**Figure 9:**
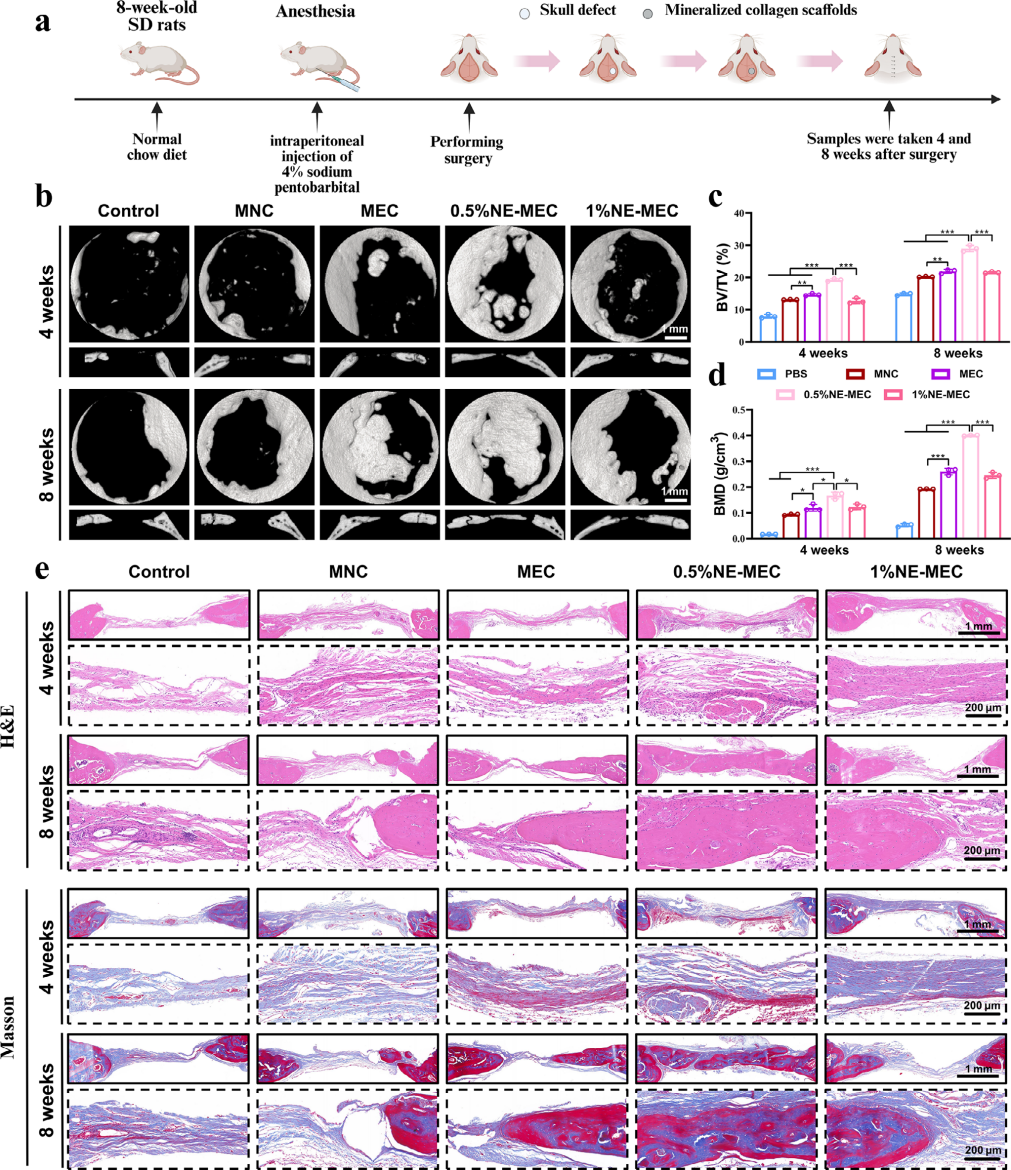
In vivo bone regeneration promoted by NE‐MEC scaffolds in a rat calvarial defect model. a) Schematic illustration of the surgical procedure for creating calvarial defects in 8‐week‐old SD rats and implanting mineralized collagen scaffolds. b) Representative 3D reconstructed micro‐CT images of calvarial defects at 4 and 8 weeks post‐implantation. Quantitative micro‐CT analysis of new bone formation, including bone volume/tissue volume (BV/TV, c) and bone mineral density (BMD, d). e Histological analysis of calvarial defect regions by H&E and Masson's trichrome staining at 4 and 8 weeks. Scale bars: 1 mm and 200 µm in (e). *P < 0.05; **P < 0.01; ***P < 0.001. a Created with biorender.com. Data are mean ± SD (n = 3).

To evaluate the therapeutic efficacy of NE‐MEC scaffolds in vivo, we implanted different scaffolds into rat critical‐sized calvarial defects and analyzed bone regeneration, neurovascularization, and immune responses at 4 and 8 weeks post‐operation, following the experimental procedure illustrated in **Figure** **9**a. Micro‐CT sagittal and coronal reconstructions of the calvarial specimens were obtained (Figure 9b), showing that new bone had formed in both the control and experimental groups at 4 and 8 weeks. The newly formed bone extended from the edges of the calvarial defects toward the center. Bone regeneration in each group was more pronounced at 8 weeks compared to 4 weeks, indicating time‐dependent repair progression. Compared to the blank control group, all four material‐implanted groups exhibited significantly increased bone volume, demonstrating that mineralized collagen scaffolds effectively promote bone defect repair. Qualitative inspection of the Micro‐CT images revealed the following trend in new bone formation: 0.5%NE‐MEC > MEC > MNC > PBS. This suggests that the mineralized collagen matrix itself—mimicking the natural bone composition—can promote in vivo bone regeneration. Moreover, MEC scaffolds outperformed MNC, likely due to their enhanced structural coherence and mechanical integrity, which may facilitate sustained HA release, guided osteoblast migration, and axonal extension. The addition of an appropriate concentration of NE further enhanced this effect. Notably, 0.5%NE‐MEC demonstrated greater regenerative efficacy than 1%NE‐MEC, suggesting a concentration‐dependent influence of NE on osteogenesis. Quantitative analysis of BV/TV and BMD confirmed the Micro‐CT observations. All scaffold‐treated groups showed significantly increased BV/TV and BMD compared to the untreated PBS control group (Figure 9c,d), with the 0.5%NE‐MEC group exhibiting the best performance.

H&E staining at 4 and 8 weeks (Figure 9e) revealed limited bone repair in the control (PBS) group, where the defect area was mainly filled with fibrous connective tissue accompanied by substantial inflammatory cell infiltration, and almost no new bone formation was observed even at 8 weeks. In contrast, all scaffold‐treated groups—MNC, MEC, 0.5%NE‐MEC, and 1%NE‐MEC—exhibited substantial new bone formation within the defect area. Consistent with Micro‐CT findings, the 0.5%NE‐MEC group showed the most extensive and continuous new bone regeneration, well‐integrated with surrounding host tissue. Masson's trichrome staining provided further support for these observations (Figure 9e). At 8 weeks, all treatment groups exhibited extensive collagen deposition and bone matrix remodeling, with the 0.5%NE‐MEC group displaying the most densely organized and mature bone tissue, along with evidence of neovascularization within the defect area. Overall, the regenerative effect followed a general trend: 0.5%NE‐MEC > MEC, which was comparable to 1%NE‐MEC > MNC > PBS. Figure S10 (Supporting Information) confirmed the absence of systemic toxicity via HE staining of major organs, validating the biocompatibility of scaffolds in vivo.

**Figure 10:**
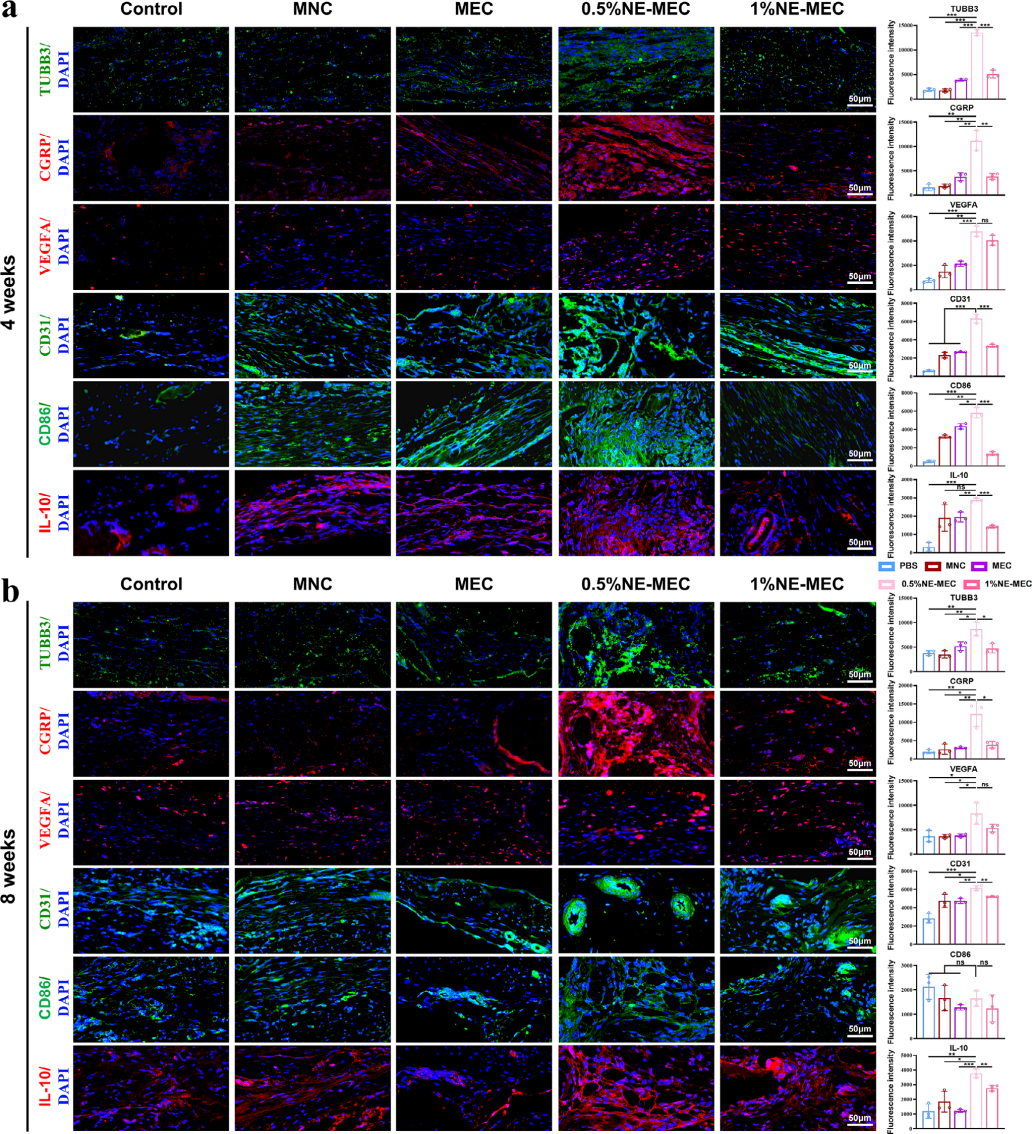
In vivo immunofluorescence analysis of neurovascular and immune responses at 4 and 8 weeks post‐implantation. Representative immunofluorescence images showing the expression of TUBB3 (neuronal marker), CGRP (sensory neuropeptide), VEGFA (angiogenic factor), CD31 (endothelial cell marker), CD86 (M1 macrophage marker), and IL‐10 (M2 macrophage marker) in bone defect regions at 4 (a) and 8 (b) weeks across different groups. Quantitative analysis of fluorescence intensity is shown on the right. Scale bars: 50 µm. *P < 0.05; **P < 0.01; ***P < 0.001; ns, not significant. Data are mean ± SD (*n* = 3).

Subsequently, immunofluorescence staining was performed on the bone tissue at the defect site to evaluate the regenerative microenvironment. NE‐MEC scaffolds exhibited significant effects on neurogenesis, angiogenesis, and immunomodulation at both 4 and 8 weeks post‐implantation. At 4 weeks (**Figure** **10**a), TUBB3 expression—a neuron‐specific marker—was elevated in all scaffold‐treated groups compared to the PBS and MNC controls, with the highest expression observed in the 0.5%NE‐MEC group, indicating enhanced neurogenesis. The pro‐neurogenic effect of the MEC group may stem from its anisotropic structure facilitating axonal alignment, while NE‐MEC likely augments this process by promoting neurotrophic factor expression in BMSCs. CGRP, a sensory neuropeptide known to support both vascularization and osteogenesis, was also most strongly expressed in the 0.5%NE‐MEC group, in line with the TUBB3 results—highlighting the key role of sensory nerves in bone repair. In addition, VEGFA expression—indicative of local angiogenic signaling—was markedly upregulated in the 0.5%NE‐MEC group, likely due in part to NE‐enhanced VEGFA expression by BMSCs, as shown in earlier transcriptomic data. Both CGRP and VEGFA are known to promote angiogenesis, and consistent with this, CD31 expression (a direct marker of vascular formation) was significantly elevated in the 0.5%NE‐MEC group. Interestingly, CD86 expression (an M1 macrophage marker) was upregulated in all scaffold‐treated groups relative to PBS, possibly due to early‐stage inflammatory activation that is beneficial for initiating tissue repair. However, the 1%NE‐MEC group exhibited notably lower CD86 expression compared to other scaffold groups, indicating a potential inhibitory effect on M1 polarization. IL‐10 expression (an M2 macrophage marker) was significantly elevated in all groups compared to PBS, with the 0.5%NE‐MEC groups showing the strongest upregulation, suggesting enhanced M2 polarization. These data indicate that during the early healing phase (week 4), the 0.5% NE‐MEC scaffold effectively stimulates neurogenesis and angiogenesis, while simultaneously modulating the immune response toward a pro‐regenerative profile favorable for bone tissue repair. By 8 weeks (Figure 10b), the 0.5%NE‐MEC group maintained high expression of both TUBB3 and CGRP, indicating sustained neurogenic activity. VEGFA and CD31 expression levels also remained elevated, reflecting continued angiogenesis and vascular maturation. These findings are consistent with prior histological observations of dense, vascularized bone matrix. In terms of immune modulation, CD86 levels did not differ significantly among groups, but IL‐10 remained elevated in the NE‐MEC groups, particularly in the 0.5%NE‐MEC condition. This likely reflects the resolution of early inflammation and the transition of macrophages from a pro‐inflammatory M1 to a reparative M2 phenotype, which facilitates later‐stage bone regeneration. In summary, these results demonstrate that the 0.5%NE‐MEC scaffold not only supports robust bone formation but also promotes coordinated neurovascular regeneration and favorable immune polarization in vivo, all of which likely act synergistically to enhance overall repair outcomes.

To assess the in vivo degradation of the scaffolds, macroscopic observations were performed at 4 and 8 weeks after implantation. As shown in Figure S11 (Supporting Information), NE‐MEC scaffolds exhibited gradual resorption with reduced visible remnants at 8 weeks, indicating effective biodegradability and integration into the host tissue. These findings further support the translational potential and biosafety of NE‐MEC.

## 3 Discussion

Repairing large bone defects remains a major clinical challenge, as it requires not only osteogenesis but also coordinated neurovascular regeneration and immune modulation. Inspired by the composition and structure of native bone tissue and its complex interplay of biological signals, we have developed a norepinephrine‐loaded biomimetic mineralized electrocompacted collagen scaffold (NE‐MEC) capable of simultaneously supporting osteogenesis, neural repair, angiogenesis, and immune modulation. In this study, we report the fabrication and characterization of a mineralized, electrocompacted collagen scaffold incorporating norepinephrine as an active factor. This NE‐MEC scaffold combines biomimetic alignment, bone‐mimicking composition, and sustained biochemical signaling to engage multiple regenerative pathways. Through a combination of in vitro assays, transcriptomic profiling, and a rat calvarial defect model, we demonstrate that the NE‐MEC scaffold promotes osteogenic differentiation, supports neurovascular regeneration, and modulates macrophage polarization toward a pro‐regenerative phenotype.

Bone tissue accounts for ≈15%–20% of total body weight in adults and serves as a mechanically active organ composed of multiple hierarchical structures capable of bearing diverse biomechanical loads.^[^ ^22^ ^]^ Weiner et al.^[^ ^23^ ^]^ were among the first to classify bone into seven hierarchical levels, ranging from its fundamental components to its gross morphology. With the advent of nanoscale 3D imaging technologies, Reznikov et al.^[^ ^24^ ^]^ further refined this classification by subdividing the fifth level into four additional categories, ultimately proposing a nine‐level hierarchy. The first four levels represent the microscale organization of mineralized collagen fibrils and their supramolecular arrangement, while the remaining five levels correspond to macroscopic structural elements such as woven and lamellar bone, osteons, trabecular and cortical bone.^[^ ^25^ ^]^ The design of our MEC scaffold was inspired by this hierarchical organization and biphasic composition of native bone. Mimicking both the structural and compositional features of bone is essential for engineering biomaterials that recapitulate its mechanical robustness and biological performance. As such, natural bone serves as a valuable blueprint for developing next‐generation scaffolds for skeletal regeneration.^[^ ^26^ ^]^ Mineralized collagen scaffolds have already demonstrated promising outcomes in the treatment of cranial, oral, and femoral defects, among others.^[^ ^27^, ^28^, ^29^ ^]^ These scaffolds offer excellent biocompatibility, osteoinductivity, and biodegradability in vivo, these factors that may explain the inherent bone‐healing capacity observed in the MEC group of our study, even in the absence of NE.^[^ ^30^ ^]^ Our data confirm that MEC scaffolds alone can significantly upregulate osteogenic markers such as Runx2 and Col1a1 in rBMSCs without the addition of exogenous growth factors. Furthermore, the aligned architecture of MEC scaffolds may enhance in vivo bone regeneration through topographical cues that facilitate stem cell adhesion, cytoskeletal reorganization, and axonal extension.^[^ ^19^, ^20^, ^21^ ^]^ The in vitro fabrication of mineralized collagen is referred to as biomimetic mineralization, a technique developed based on natural biomineralization.^[^ ^31^, ^32^ ^]^ Biomineralization describes the process by which organisms, under the regulation of macromolecules such as proteins, selectively deposit environmental ions (e.g., Ca^2^⁺, PO_4_^3^^−^, CO_3_^2^^−^) to form inorganic minerals. This term encompasses both the mineralization process and its products, such as bones, teeth, and shells.^[^ ^32^, ^33^ ^]^ Biomimetic mineralization strategies are generally categorized into extrafibrillar and intrafibrillar approaches.^[^ ^34^, ^35^, ^36^ ^]^ In natural bone tissue, inorganic minerals are distributed both on the surface and within the gaps of collagen fibrils. The ordered intrafibrillar mineralization is critical for achieving the mechanical properties of bone.^[^ ^35^ ^]^ Studies have shown that the absence of intrafibrillar minerals leads to increased bone brittleness.^[^ ^36^ ^]^ Therefore, mimicking intrafibrillar mineralization in bone repair materials is highly attractive for the development of advanced composites with complex morphology and superior mechanical properties, as well as for gaining deeper insights into the mechanisms of biomineralization. In this study, we used PAA‐stabilized amorphous ACP as a precursor to achieve intrafibrillar mineralization, in combination with electrochemical isoelectric focusing and uniaxial stretching. The resulting MEC scaffold features anisotropic, aligned collagen fibrils densely mineralized from within, preserving the D‐periodicity of collagen. This anisotropic structure not only enhances the mechanical strength of the scaffold, as reflected by the increased tensile modulus along the axis of alignment, but also plays a critical role in directing cellular behavior. Specifically, the aligned mineralized collagen fibrils facilitate the oriented adhesion of rBMSCs, promote the organized arrangement of the cytoskeleton, and contribute to improved bone repair outcomes in rats. Additionally, these topographical features support the directional extension of neuronal axons, which is beneficial for neural regeneration. This bone‐inspired design highlights the importance of structural and compositional biomimicry in bone tissue engineering.

In this study, NE is the only exogenous bioactive factor incorporated into the NE‐MEC composite scaffold. A comprehensive understanding of its biological function and mechanism of action is essential to elucidate its contribution to bone regeneration. As a classic sympathetic neurotransmitter, NE is abundantly distributed in sympathetic nerve terminals within the skeletal system, and its role in regulating bone homeostasis has received increasing attention in recent years.^[^ ^2^, ^3^ ^]^ However, there is ongoing controversy regarding whether NE promotes or inhibits osteogenesis. Some studies report that NE may act through β‐adrenergic receptors (β‐AR), which are widely expressed in bone tissue.^[^ ^2^ ^]^ Specifically, NE was shown to inhibit human BMSC (hBMSC) proliferation by activating β2‐AR and inducing phosphorylation of ERK1/2 and PKA.^[^ ^37^ ^]^ Additionally, NE has been implicated in directly promoting osteoclastogenesis via ROS production, a process that can be inhibited by β‐AR blockers such as propranolol.^[^ ^38^, ^39^ ^]^ In contrast, one study reported that NE can stimulate DNA synthesis and promote proliferation in rBMSCs via α1‐adrenergic receptors (α1‐AR) and downstream Ca^2^⁺/PKC and ERK1/2 signaling pathways.^[^ ^12^ ^]^ We speculate that two major factors contribute to these discrepancies. First, different studies employed different cell types, which can lead to substantial biological variation. Second, NE acts through two classes of receptors: α‐AR and β‐AR, both of which are expressed across various bone‐related cells including BMSC.^[^ ^12^, ^37^, ^40^, ^41^, ^42^, ^43^ ^]^ During bone regeneration, the effects of NE are concentration‐dependent: at lower concentrations, NE preferentially binds to α‐AR, while at higher concentrations, β‐AR‐mediated pathways dominate.^[^ ^44^, ^45^ ^]^ Therefore, it is plausible that differences in NE concentration account for conflicting outcomes in the literature. Our study provides new evidence supporting the beneficial effects of low‐dose NE (specifically 0.5%NE‐MEC) in bone tissue engineering (rat model). Scaffolds incorporating 0.5% NE significantly upregulated key osteogenic transcription factors such as Runx2 and Col1a1 in rBMSCs, and exhibited the highest ALP activity and calcium nodule formation. In contrast, a higher concentration (5%NE‐MEC) was found to inhibit both rBMSC proliferation and osteogenic differentiation.

A particularly noteworthy finding in this study is that NE significantly induced early (day 3 and 7) upregulation of NGF in rBMSCs. Previous research has shown that NGF is predominantly expressed during the early stages of bone injury repair and plays a critical role in the neuro‐osteogenic interplay.^[^ ^6^ ^]^ The same study indicated that NGF in newly formed callus originates mainly from periosteal mesenchymal cells and CD45⁺ inflammatory cells.^[^ ^6^ ^]^ Our findings are consistent with this, suggesting that NE may enhance early NGF expression in rBMSCs, thereby fostering a microenvironment that integrates neural, skeletal, and vascular cues to facilitate bone regeneration. In addition, a study demonstrated that NE exhibits a positive role in immune regulation by promoting macrophage polarization toward anti‐inflammatory and tissue‐reparative phenotypes.^[^ ^14^ ^]^ This immunomodulatory capability provides a theoretical basis for the potential of NE in tissue regeneration. Moreover, mineralized collagen itself has been reported to regulate macrophage polarization and thereby improve the osteoimmune microenvironment, ultimately promoting bone regeneration.^[^ ^46^ ^]^ Although the biomedical use of NE is still in its early stages, being limited by its short half‐life and rapid metabolism, advances in materials science and delivery strategies such as scaffold‐based encapsulation offer promising solutions to harness its full therapeutic potential.

Although the NE‐MEC scaffold did not exhibit significant pro‐angiogenic effects in in vitro experiments, it demonstrated pronounced enhancement of neovascularization in vivo, highlighting the substantial differences between in vitro and in vivo models. Similarly, some variations in macrophage polarization were also observed between in vitro and in vivo experiments, albeit to a lesser extent. One of the main reasons for this discrepancy may be the complex nature of the in vivo microenvironment. In vitro culture conditions lack various physiological signals such as immune cell interactions and neural signals, which collectively contribute to a highly dynamic regenerative niche in vivo.^[^ ^47^ ^]^ In this study, we observed that while the NE‐MEC scaffold had limited direct effects on endothelial cell migration and tube formation in vitro, it significantly upregulated VEGFA, CGRP, and CD31 expression in vivo. This suggests that the scaffold's pro‐angiogenic effects in vivo may not rely solely on direct stimulation of endothelial cells but are instead mediated indirectly through the VEGFA, as well as through the NGF–CGRP signaling axis that influences endothelial activity. However, it is important to note that BMSCs represent only one source of VEGFA and NGF, and other angiogenic factors and signaling pathways may also play critical roles. Moreover, there is a well‐established functional interplay between the nervous and vascular systems. During the early stages of bone healing, neural regeneration typically precedes angiogenesis and facilitates vascular growth by releasing neuropeptides such as CGRP.^[^ ^6^, ^7^ ^]^ These complex multisystem interactions are difficult to replicate in vitro, which partially explains the discrepancy between the scaffold's limited pro‐angiogenic activity in vitro and its significant vascularization‐promoting effects in vivo.

Scaffolds based on NGF or conductive polymers generally act through a single pathway, either by delivering NGF^[^ ^4^ ^]^ or by providing electrical stimulation.^[^ ^48^ ^]^ In contrast, our NE‐MEC scaffold not only enables controlled NE release but also establishes a regenerative circuit through the interaction between nerves and BMSCs, thereby coordinating osteogenesis, neurogenesis, angiogenesis, and immunomodulation simultaneously. Looking ahead, further studies are needed to optimize scaffold degradation kinetics, refine NE delivery strategies, and evaluate long‐term biosafety in large‐animal models. With these advances, the NE‐MEC scaffold holds promise as a translational platform for the integrated repair of critical‐sized bone defects and potentially other complex tissue injuries that require simultaneous neurovascular, osteogenic, and immunomodulatory regulation.

Despite the promising results achieved in this study, several limitations warrant consideration. First, the regenerative efficacy of the NE‐MEC scaffold has thus far been demonstrated only in a rat calvarial defect model; further validation in other species and more complex or large‐animal models is necessary to assess its translational potential. Second, this study primarily focused on the response of rBMSCs, while the roles and interactions of other critical cell types, such as glial cells, were not thoroughly explored. Third, as NE may act through multiple receptor subtypes and downstream signaling pathways, the current mechanistic understanding remains preliminary and requires further investigation. Fourth, the precise mechanisms by which CGRP mediates osteogenesis and angiogenesis, as well as the functional consequences of scaffold‐induced immune modulation, remain insufficiently explored.

## 4 Experimental Section

### Preparation of Materials

Amorphous calcium phosphate (ACP) solution was prepared using a polymer‐stabilized method. First, 0.05 g of polyacrylic acid (PAA) was dissolved in 40 mL of deionized (DI) water and stirred at room temperature for 15 min. Then, 0.222, 0.444, or 0.888 g of anhydrous calcium chloride (CaCl_2_) was added, and the mixture was stirred until homogeneous. In a separate beaker, 0.05 g of PAA was dissolved in 40 mL of DI water, followed by the addition of potassium hydrogen phosphate (K_2_HPO_4_·3H_2_O) to prepare a 0.03 m, 0.06 m, or 0.12 m K_2_HPO_4_ solution (corresponding to 25 mm ACP, 50 mm ACP, or 100 mm ACP). This solution was slowly added dropwise to the CaCl_2_ solution under continuous stirring. After the addition, the mixture was stirred for 30 min at room temperature to ensure uniform ion distribution and amorphous phase stability. The final Ca:P ratio was adjusted to 1.67 by adding NaOH to raise the pH to 9.5.

Fresh bovine tendons (50 g) were first cleaned by removing the tendon sheath, fat, and muscle, and soaked in 10% NaCl solution for 24 h. After multiple washes with deionized water, the tendons were air‐dried at room temperature. The tendons were then immersed in 0.5 m acetic acid for 2 h and enzymatically digested with pepsin at a ratio of 1:50 (pepsin: tendon weight) at 4 °C for 72 h under constant stirring. After digestion, the solution was filtered to remove solid fragments, and the filtrate was centrifuged at 4000 rpm for 20 min. The supernatant was collected, followed by salting out with 0.75 m NaCl solution, and dialyzed at 4 °C for 3 days. The collagen solution was then freeze‐dried, and the final concentration of collagen was determined. The purified collagen was dissolved in DI water, to a final concentration of 0.75 mg mL^−1^ to prepare a total of 20 mL collagen solution. NE was then added to the collagen solution at different weight ratios: 0.5%, 1%, and 5% (w/w), corresponding to 5, 10, and 50 µL of a 20 mg mL^−1^ NE solution, respectively. The mixture was stirred for 30 min to ensure thorough mixing. A titanium (Ti) sheet was used as the anode, and a graphite sheet as the cathode. A constant voltage of 15 V was applied to the collagen solution for 15 min using an electrochemical workstation, resulting in the formation of densely aligned collagen sheets. To obtain scaffolds with suitable thickness, multiple rounds of electrocompaction were performed sequentially. The collagen sheets were then subjected to stretching at a rate of 2 mm min^−1^ to achieve 150% elongation, further enhancing the directional alignment of the collagen fibers. The stretched collagen sheets were immersed in ACP solutions with varying calcium ion concentrations at 37 °C for 12 h. After mineralization, the materials were freeze‐dried to obtain NE‐loaded, anisotropic mineralized collagen scaffolds. Electrocompacted collagen sheets are referred to as EC, while collagen sheets prepared without electrocompacted alignment are denoted as NC. Upon mineralization in ACP solutions (unless otherwise specified, 50 mm ACP was used as the default condition), EC and NC scaffolds were designated as MEC and MNC, respectively. When NE was incorporated into the MEC scaffold, the resulting composite is referred to as NE‐MEC. The physical image of the NE‐MEC scaffold is shown in Figure S1 (Supporting Information).

### Structural and Mechanical Characterization

The surface and cross sectional morphologies of mineralized collagen scaffolds were observed using field‐emission scanning electron microscopy (FE‐SEM, JSM‐7800F Prime, JEOL, Japan) following gold sputter coating. Ultrastructural features, including fibril organization and mineral deposition, were further examined by transmission electron microscopy (TEM, JSM 2100F, JEOL, Japan) after resin embedding, ultramicrotomy, and staining. The phase composition and crystallographic structure of the scaffolds were analyzed using X‐ray diffraction (XRD, Empyrean, Malvern Panalytical, Netherlands) with Cu‐Kα radiation over a 2θ range of 5–80°. Fourier‐transform infrared spectroscopy (FTIR, Nicolet iS50, Thermo, USA) was conducted on KBr‐pelletized samples to assess the chemical bonds and structural changes in the mineralized collagen scaffolds. The mechanical properties of scaffolds were evaluated by uniaxial tensile testing at a constant strain rate of 2 mm min^−1^ using rectangular specimens (2.5 cm × 2.5 cm × 1 mm), and stress–strain curves were recorded for analysis.

### Swelling Behavior Assessment

The swelling behavior of the mineralized collagen scaffolds was evaluated using an analytical electronic balance to measure the weight change over time. The initial dry weight of the scaffolds (Wd) was recorded. The scaffolds were then immersed in PBS buffer solution (pH 7.4) at 37 °C, ensuring the samples were fully submerged. At designated time points, the samples were removed from the solution, and the surface water was carefully blotted dry using filter paper. The swollen weight of the samples (Ws) was then recorded. The swelling ratio (SR) was calculated using the following equation:

(1) $$\mathrm{SR}\left(\%\right)=\left(W_{s}-W_{d}\right)/W_{d}\times 100\%$$

### Thermogravimetric Analysis (TGA)

The mineral content of various scaffolds was determined using TG Analysis (TGA/DSC3+, METTLER TOLEDO, Switzerland). The analysis was performed in air over a temperature range from room temperature to 700 °C at a heating rate of 5 °C min^−1^. This value reflects the proportion of inorganic minerals remaining after thermal decomposition of the organic matrix. The calculation was performed using the following equation:

(2)

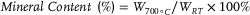




### Isolation and Culture of Primary Cells

Rat bone marrow mesenchymal stem cells (rBMSCs) were isolated from the femurs and tibias of 2–3‐week‐old male Sprague‐Dawley (SD) rats under sterile conditions. Bone marrow was flushed and filtered through a 70 µm cell strainer, then cultured in α‐MEM (Gibco, USA) supplemented with 10% fetal bovine serum (FBS) and 1% penicillin‐streptomycin (PS). Cells were maintained at 37 °C in a humidified incubator with 5% CO_2_, with medium changes every 2–3 days. Upon reaching 80–90% confluence, cells were passaged at a 1:3 ratio using 0.25% trypsin‐EDTA. Only passages 3–5 were used for experiments. For scaffold experiments, 2 mg of material was added per well in 6‐well plates, and 1 mg per well for 12‐well plates.

Dorsal root ganglion (DRG) neurons were obtained from 1–2‐week‐old SD rats. After euthanasia and surface sterilization, DRGs were carefully dissected and incubated sequentially with 1.25 g L^−1^ Type I collagenase (60 min, 37 °C) and 2.5 g L^−1^ trypsin‐EDTA (20 min, 37 °C). Enzymatic digestion was terminated with complete neuronal medium (DMEM containing 2% horse serum, 1% PS, and 100 ng mL^−1^ NGF). After filtration (100 µm) and centrifugation, the cells were resuspended and seeded onto poly‐L‐lysine (20 µg mL^−1^, 1 h at 37 °C) and laminin (10 µg mL^−1^, overnight at 4 °C) coated plates. The cells were incubated at 37 °C with 5% CO_2_ for 30 min to allow for differential attachment. Fibroblasts and glial cells typically adhered faster than the neurons, and non‐adherent cells were carefully collected by aspirating the supernatant. The aspirated medium was transferred to another culture dish and incubated at 37 °C with 5% CO_2_. After overnight incubation, half of the culture medium was replaced. Subsequently, the culture medium was changed every 3 days, with half of the volume replaced to maintain healthy neuronal cultures.

### Culture of Additional Cell Lines

PC12 cells, RAW264.7 macrophages, and human umbilical vein endothelial cells (HUVECs) were also used in this study. PC12 cells were maintained in RPMI‐1640 medium supplemented with 10% FBS and 1% PS. For differentiation, the medium was replaced with RPMI‐1640 containing 1% horse serum and 1% PS. RAW264.7 cells were cultured in DMEM with 10% FBS and 1% PS. HUVECs were maintained in DMEM supplemented with 10% FBS and 1% PS during expansion, while serum‐free medium was used in functional assays such as tube formation and wound healing. All cell lines were incubated at 37 °C in a humidified atmosphere containing 5% CO_2_.

### Cytocompatibility Assessment

Cell viability and cytotoxicity were assessed using the Calcein/PI Cell Viability and Cytotoxicity Assay Kit (C2015M, Beyotime, China) following the manufacturer's instructions. After treatment, cells were incubated with a staining solution containing Calcein‐AM and Propidium Iodide (PI) at 37 °C for the recommended duration. Stained cells were observed under a fluorescence microscope, where live cells emitted green fluorescence, and dead cells emitted red fluorescence. Cell proliferation was evaluated using the Cell Counting Kit‐8 (CCK‐8, BS350B, Biosharp, China) according to the manufacturer's instructions. After treatment, 10 µL of the CCK‐8 solution was added to each well of the 96‐well plate, followed by incubation at 37 °C for 2 h. The absorbance was measured at 450 nm using a microplate reader to quantify cell viability and proliferation.

### Enzyme‐Linked Immunosorbent Assay (ELISA)

NE‐loaded scaffolds were placed into 50 mL centrifuge tubes containing 50 mL of PBS, either with or without 2 U mL^−1^ collagenase. The tubes were incubated at 37 °C in the dark and shaken at 40 rpm. At designated time points, 100 µL of the PBS solution was collected for analysis. Since the sampling volume accounted for only 0.2% of the total volume, no additional compensation was made for the sampled liquid. The NE concentration in the collected samples was measured using a Rat NE ELISA Kit (JRX302393R, RUIXIN BIOTECH, China) according to the manufacturer's instructions.

### RNA Sequencing and Transcriptomic Analysis

To investigate the molecular effects of NE and NE‐functionalized scaffolds on rBMSCs, two independent RNA‐sequencing (RNA‐seq) experiments were conducted. In the first experiment, rBMSCs were treated with 10^−6^ m NE for 24 h under standard culture conditions to explore NE‐specific gene regulation. In the second experiment, rBMSCs were co‐cultured with either MEC or 0.5% NE‐loaded MEC (0.5%NE‐MEC) scaffolds in osteogenic induction medium (DMEM supplemented with 10% FBS, 1% PS, 50 µg mL^−1^ ascorbic acid, 0.1 µm dexamethasone, and 5 mm β‐glycerophosphate) for 7 days to assess scaffold‐mediated transcriptomic changes.

Total RNA was extracted from all samples and submitted to OE Biotech Co., Ltd. (Shanghai, China) for sequencing. RNA quality and concentration were assessed prior to library construction using the NEBNext Ultra II RNA Library Prep Kit, followed by purification with the AMPure XP system (Beckman Coulter, USA). Libraries were sequenced on the Illumina NovaSeq 6000 platform. Bioinformatic analyses, including differential expression analysis and functional enrichment (GO, KEGG), were performed using the OECloud online platform (https://cloud.oebiotech.com/task/). Data visualization, such as volcano plots and heatmaps, was generated using R‐based modules integrated into the OECloud suite. To validate the transcriptomic findings at the protein level, several key osteogenic genes identified in the RNA‐sequencing analysis—including RUNX2 (Proteintech, 20700‐1‐AP), ALP (SCT, sc‐365765), and Col1a1 (Proteintech, 67288‐1‐Ig)—were further confirmed by Western blot (WB) analysis. GAPDH (Proteintech, 60004‐1‐Ig) was used as the internal loading control.

### In Vitro Functional Assays

To comprehensively evaluate the multifunctional effects of the developed scaffolds, a series of in vitro experiments were performed to assess their influence on osteogenesis, neurogenesis, angiogenesis, and immunomodulation.

To investigate the scaffold's osteoinductive capacity, rBMSCs were cultured on different scaffolds in osteogenic induction medium for 7 or 14 days. Early osteogenic differentiation was assessed on day 7 by ALP staining using a BCIP/NBT kit (Beyotime, C3206). Quantitative ALP activity was determined using p‐nitrophenyl phosphate (pNPP) as a substrate, which produces a yellow product, p‐nitrophenol (PNP), with maximum absorbance at 405 nm. Late‐stage mineralization was evaluated on day 14 by Alizarin Red S (ARS) staining with a commercial osteogenesis assay kit (Beyotime, C0148S); the bound dye was solubilized with cetylpyridinium chloride and quantified at 562 nm. To further confirm osteogenic commitment, total RNA was extracted at days 7 and 14, and the mRNA levels of key osteogenic markers (*Runx2*, *ALP*, *OCN*) were quantified by qPCR. Primer sequences are listed in Table S1 (Supporting Information). Additionally, rBMSCs were co‐cultured with scaffolds for 24 h and subsequently stained with TRITC Phalloidin (Solarbio, CA1610) and anti‐vinculin antibody (Abcam, ab129002) to assess cytoskeletal organization and focal adhesion formation.

To assess scaffold‐mediated neurogenic effects, both indirect and direct co‐culture models with DRG neurons were established. In the indirect model, rBMSCs were co‐cultured with scaffolds for 3 days. Then, the supernatant was collected, centrifuged, and filtered to remove debris, then concentrated using ultrafiltration columns (Millipore, USA). The concentrated supernatants were then added to α‐MEM supplemented with 2% horse serum and 1% PS to generate the conditioned medium, which was subsequently used to culture DRG neurons for up to 6 days. Neurite outgrowth was evaluated on day 3 via TUBB3 (Proteintech, 66375‐1‐Ig, 1:500) immunofluorescence staining, while CGRP (Bioss, bs‐10639R, 1:500) expression was assessed on day 6. In addition, CGRP levels in the neuronal culture supernatants were quantified by ELISA (RX302583R, RUIXIN BIOTECH, China) to evaluate functional peptide (CGRP) secretion. In the direct co‐culture model, DRG neurons were seeded directly onto the scaffolds placed in low‐adhesion plates and maintained for 4 days in NGF‐supplemented medium. Neuronal axonal orientation along the scaffold topography were visualized using TUBB3 staining to evaluate material‐guided neurite alignment. Meanwhile, to explore the scaffold's potential to induce neurotrophic support, expression of *NGF* in rBMSCs was analyzed by qPCR on days 3, 7, and 14.

Angiogenic potential was evaluated using HUVECs cultured in scaffold‐conditioned serum‐free medium. Tube formation assays were performed on Matrigel‐coated plates, and angiogenic capacity was quantified by measuring total tube length and the number of junctions. In parallel, wound healing assays were conducted to assess endothelial cell migration across a scratch under serum‐free conditions. Immunomodulatory effects were examined by culturing RAW264.7 macrophages with scaffolds for 24 h. Cells were then fixed and subjected to immunofluorescence staining for macrophage polarization markers. M1‐associated markers included CD86 (Proteintech, 13395‐1‐AP) and TNF‐α (Proteintech, 60291‐1‐Ig), while M2‐associated markers included CD206 (Proteintech, 18704‐1‐AP) and IL‐10 (Proteintech, 60269‐1‐Ig). To further quantify macrophage polarization‐related cytokines, the levels of TNF‐α and IL‐10 in the culture supernatants were measured using ELISA kits (RX202412M and RX203075M, RUIXIN BIOTECH, China) according to the manufacturer's instructions.

### In Vivo Animal Studies

SD rats were purchased from Chengdu Dashuo Animal Co., Ltd. Animal experiments were performed on 8‐week‐old rats. A total of *n* = 3 animals were used per group. Animals were randomly assigned to each experimental group. All surgical procedures were performed by investigators blinded to the group allocation. Anesthesia was induced via an intraperitoneal injection of 4% sodium pentobarbital (40 mg kg^−1^). A 2 cm incision was made along the sagittal suture (the midline of the skull) using a surgical scalpel to expose the underlying skull. Under continuous irrigation with sterile saline, a 5 mm diameter cranial defect was created unilaterally, ≈4 mm to the right of the sagittal suture, specifically on the right parietal bone, using a trephine drill. The 5 mm defect is commonly considered critical‐sized in rat calvarial models due to its limited self‐healing ability, though some controversy exists across different experimental designs.^[^ ^49^, ^50^ ^]^ The prepared scaffolds were implanted into the defect sites on the right parietal bone, and the skin was sutured closed. After 4 and 8 weeks, the rats were euthanized via intraperitoneal injection of an overdose of sodium pentobarbital. The skulls were harvested and fixed in 4% paraformaldehyde.

The harvested skull samples were fixed in 4% paraformaldehyde and scanned using a micro‐computed tomography (Micro‐CT) system (SCANCO Medical, Bruettisellen, Switzerland). The scanning parameters were set to a resolution of 6 µm, with a voltage of 50 kV and current of 200 µA. 3D reconstructions were generated, and quantitative analysis was performed to assess bone volume (BV), tissue volume (TV), bone volume fraction (BV/TV), and bone mineral density (BMD) within the defect area.

After Micro‐CT, the samples were decalcified in 10% EDTA (pH 7.4), embedded in paraffin, and sectioned at 5 µm thickness. Hematoxylin and eosin (H&E) staining was used to examine general tissue morphology, while Masson's trichrome staining (Beyotime, C0189S) was performed to evaluate collagen deposition.

For immunofluorescence analysis, tissue sections were deparaffinized, rehydrated, and subjected to antigen retrieval in citrate buffer (pH 6.0), followed by blocking with 5% bovine serum albumin (BSA). Sections were incubated overnight at 4 °C with primary antibodies against TUBB3 (Proteintech, 66375‐1‐Ig, 1:500), CGRP (Bioss, bs‐10639R, 1:500), VEGFA (Abcam, ab39250, 1:300), CD31 (Proteintech, 28083‐1‐AP, 1:500), CD86 (Proteintech, 13395‐1‐AP, 1:500), and IL‐10 (Proteintech, 60269‐1‐Ig, 1:500). After washing, appropriate fluorophore‐conjugated secondary antibodies were applied for 1 h at room temperature in the dark. Nuclei were counterstained with DAPI, and fluorescence images were captured using a confocal microscope.

All animal experiments were performed in accordance with institutional ethical guidelines and approved protocols, and the study was conducted in compliance with the ARRIVE guidelines.

### Compliance with Ethical Standards

All rat‐related procedures were approved by the Medical Ethics Committee of Southwest Jiaotong University (Approval No.: SWJTU‐2403‐NSFC‐053).

### Statistical Analysis

All experiments were conducted in triplicate (*n* = 3), and the results were presented as mean ± standard deviation (SD). Statistical analyses were performed using GraphPad Prism 9.0 software (GraphPad Software, USA). For comparisons between two groups, Student's t‐test was applied. For comparisons among three or more groups, one‐way ANOVA followed by Tukey's post hoc test was used. Normality and homogeneity of variance were assessed using the Shapiro–Wilk test and Levene's test, respectively. Statistical significance levels were set as follows: * (*P* < 0.05), ** (*P* < 0.01), *** (*P* < 0.001) and ns (not significant). Quantitative image analysis was performed using ImageJ software.

## Conflict of Interest

The authors declare no conflict of interest.

## Author Contributions

R.Z.Y. contributed to the conception and design of the study, performed experiments, and drafted and critically revised the manuscript. W.Z.J. and W.A.H. contributed to the analysis and interpretation of data, conducted animal experiments and prepared materials, and participated in critically revising the manuscript. Z.H. and L.J.C. contributed to animal experiments and critically revised the manuscript. Z.J.H.: Contributed to the preparation of collagen materials and critically revised the manuscript. W.J. contributed to interpretation and critically revised the manuscript. C.S., T.H., and G.T.L. contributed to conception, design, drafted and critically revised the manuscript. All authors gave their final approval and agree to be accountable for all aspects of the work.

## Supporting information



Supporting Information

Supplemental Data

## Data Availability

The sequencing data have been deposited in the Sequence Read Archive (SRA) database under the bioproject number PRJNA1272144 and PRJNA1271974.
