# Supplementary material for: A Biomimetic Norepinephrine‐Loaded Aligned Mineralized Collagen Scaffold for Coordinated Neurovascular, Osteogenic, and Immunomodulatory Repair of Critical‐Sized Bone Defects
Source: Adv Sci (Weinh). 2025 Nov 21;13(8):e18807. doi: 10.1002/advs.202518807 (PMC12884768; doi:10.1002/advs.202518807)
Supplement: Supplementary file 1 — Supporting Information [file ADVS-13-e18807-s001.pdf]

Supplementary Information for

A biomimetic norepinephrine-loaded aligned mineralized collagen scaffold for coordinated neurovascular, osteogenic, and immunomodulatory repair of bone defects

Zhengyun Ren [1,2,3], Zhaojun Wu [1,2], Anhang Wu [1], Hui Zhang [1,2,3], Jiachen Lu [1,2], Jiahao Zhang [1,2], Jie Weng [1], Jinhua Zhang [1,2], Song Chen[4*], Huan Tan[1*], Tailin Guo[1*]

Author affiliations:

[1] College of Medicine, Southwest Jiaotong University, Chengdu, China.

[2] Key Laboratory of Advanced Technologies of Materials Ministry of Education, School of Materials Science and Engineering, Southwest Jiaotong University, Chengdu, China.

[3] Obesity and Metabolism Medicine-Engineering Integration Laboratory, Department of General Surgery, The Third People's Hospital of Chengdu, Affiliated Hospital of Southwest Jiaotong University, Chengdu, China.

[4] Department of Orthopaedics, The General Hospital of Western Theater Command.

Corresponding author: Song Chen, Huan Tan, Tailin Guo

Email address: chensongchinese@163.com, tanhuan@swjtu.edu.cn, tlguo@home.swjtu.edu.cn

This PDF file includes:

Figure. S1-S11

Table S1

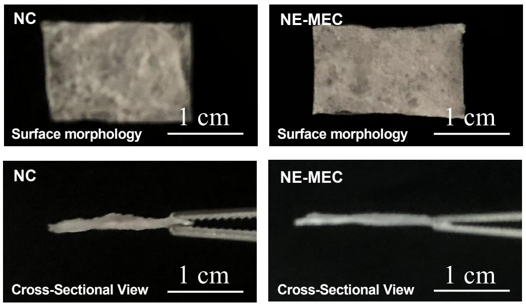

Supplementary Fig. 1| Surface morphology and cross-sectional view of the NC and NE-MEC scaffolds. Scale bars represent 1 cm.

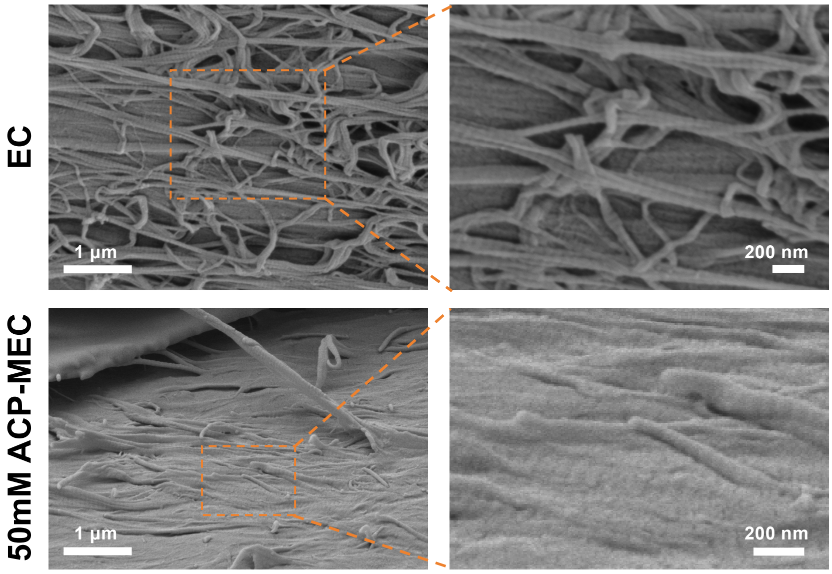

Supplementary Fig. 2| High-magnification SEM images showing the surface morphology of electrochemically aligned collagen (EC, up) and 50 mM ACP-mineralized electrochemically aligned collagen scaffold (50 mM ACP-MEC, down).

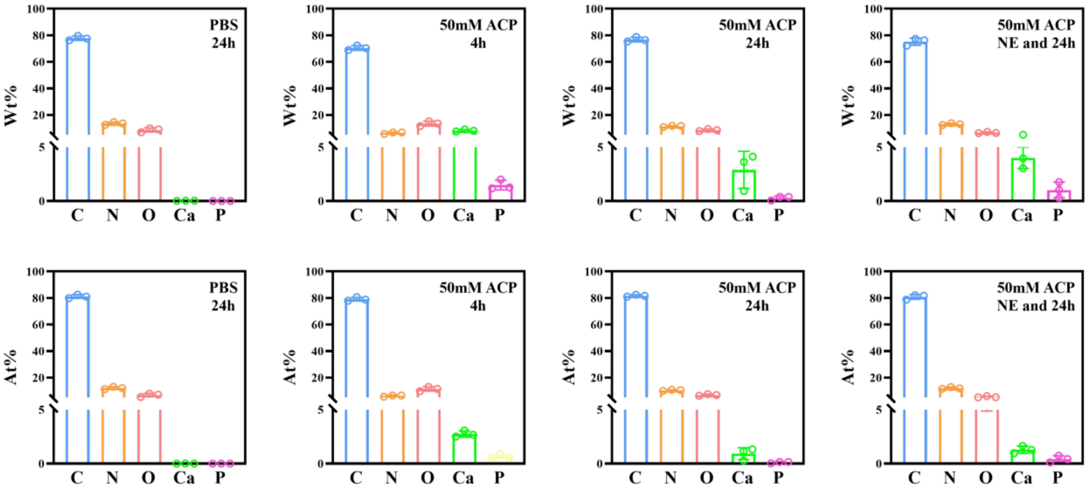

Supplementary Fig. 3| Weight percent (Wt%) and atomic percent (At%) of elemental composition for scaffold.

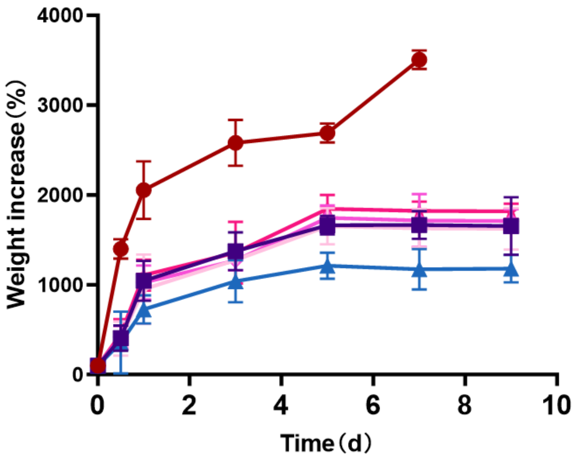

Supplementary Fig. 4| Swelling behavior over time in PBS at 37°C.

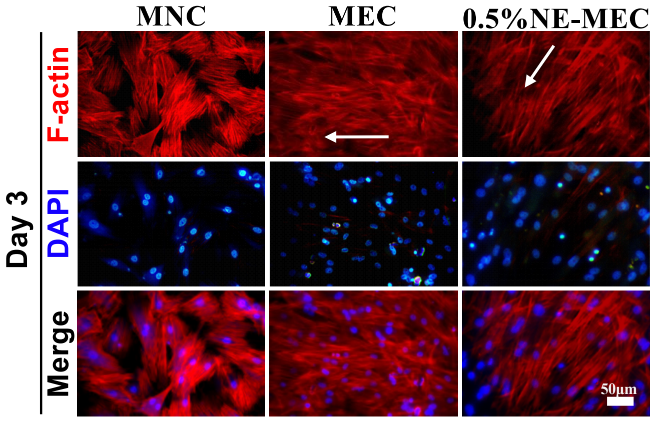

Supplementary Fig. 5| Immunofluorescence staining of rBMSCs cultured on MNC, MEC, and 0.5%NE-MEC scaffolds for 3 days in low-adhesion plates. Cells were stained for F-actin (red) and nuclei (DAPI, blue) to visualize cytoskeletal organization and cell orientation. Scale bars: 50 μm.

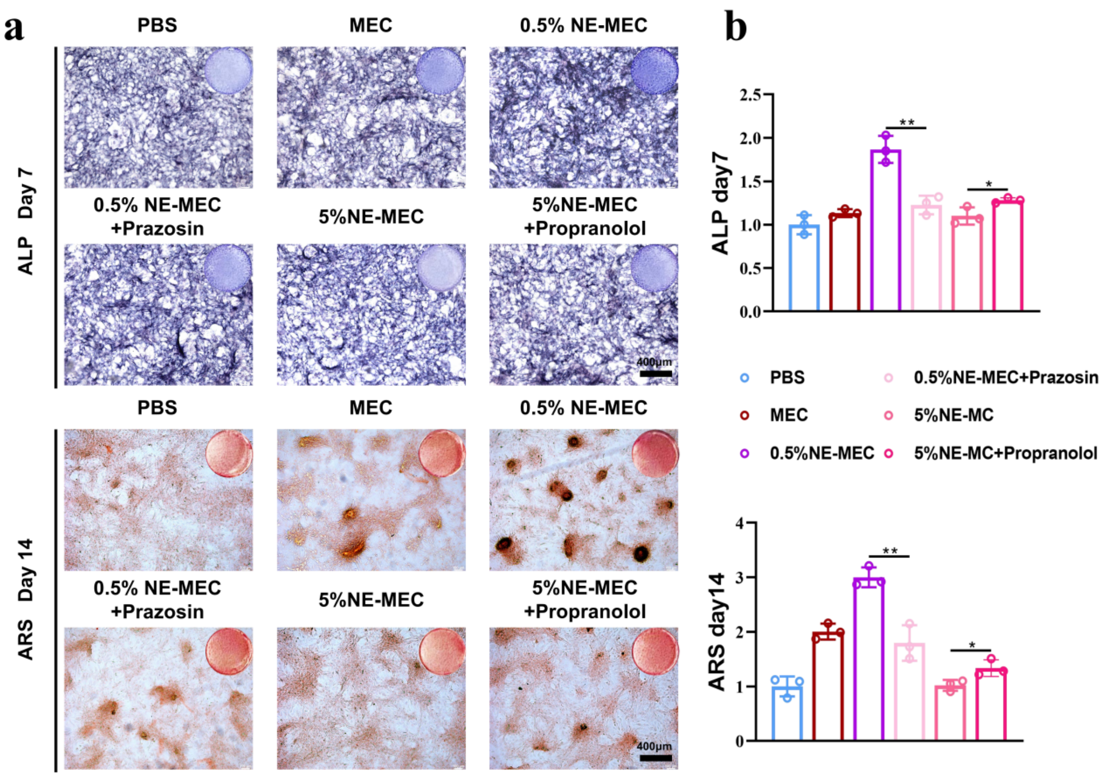

Supplementary Fig. 6| a Representative image of ALP staining on day 7 and ARS staining on day 14. **b** Quantification of ALP activity and ARS mineralization. Scale bars: 400 μm. **P* < 0.05; ***P* < 0.01. Data are mean ± SD (n = 3).

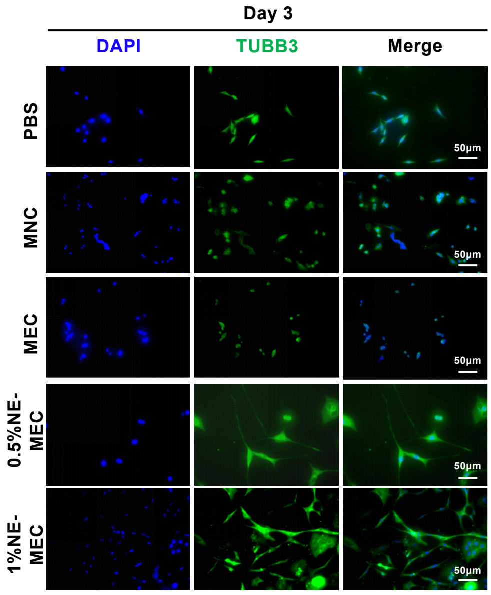

Supplementary Fig. 7| Immunofluorescence images of PC12 cells cultured in conditioned media derived from rBMSCs co-cultured with different scaffolds. Cells were stained for neuronal marker TUBB3 (green) and nuclei (DAPI, blue). Scale bars: 50 μm.

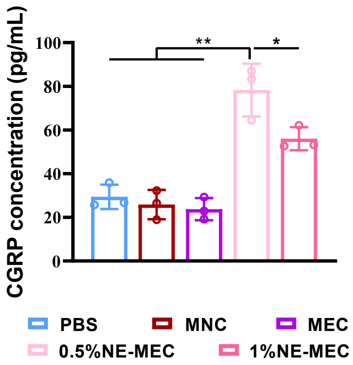

Supplementary Fig. 8| Quantification of CGRP secretion in the supernatants of neuron cultures after 6 days, measured by ELISA. Data are presented as mean ± SD (n = 3). *$P < 0.05$, **$P < 0.01$, ***$P < 0.001$.

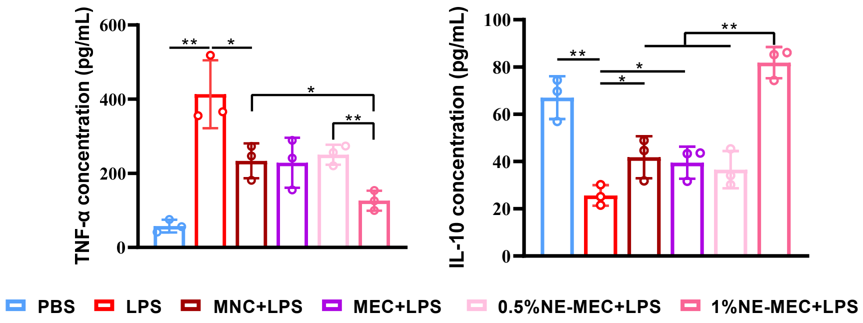

Supplementary Fig. 9| Quantitative analysis of macrophage polarization-related cytokines (TNF-α and IL-10). Data are presented as mean ± SD (n = 3). **P* < 0.05, ***P* < 0.01.

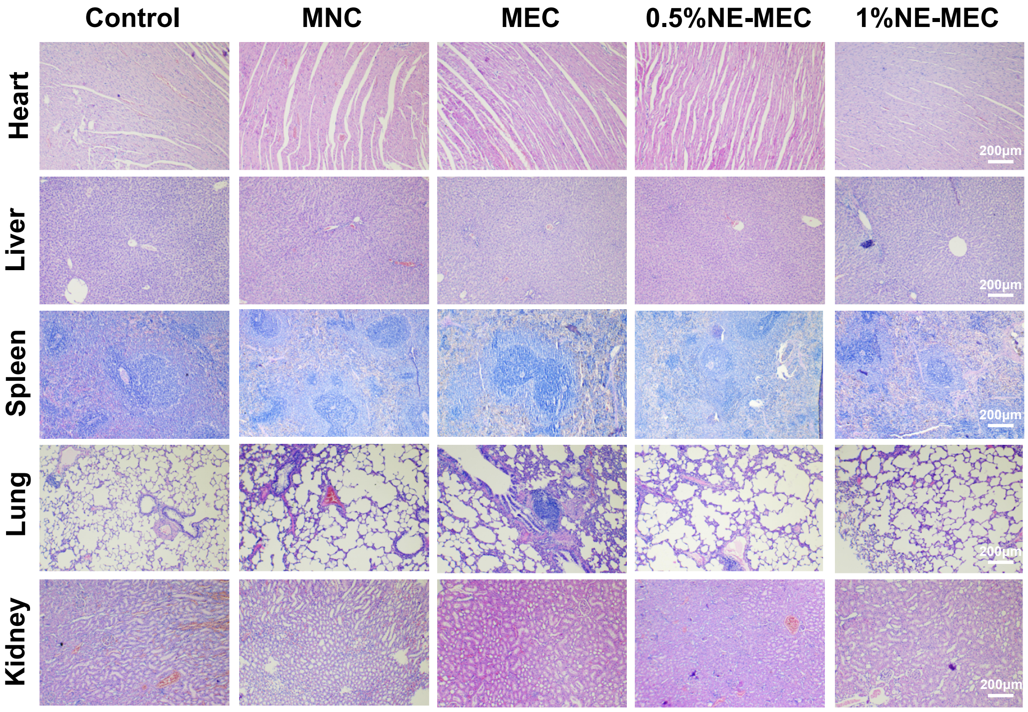

Supplementary Fig. 10| Histological evaluations of major organs to assess systemic toxicity following scaffold implantation. Hematoxylin and eosin (H&E) staining of heart, liver, spleen, lung, and kidney tissues harvested from rats in different groups (Control, MNC, MEC, 0.5%NE-MEC, 1%NE-MEC) at 8 weeks post-implantation. Scale bars: 200 μm.

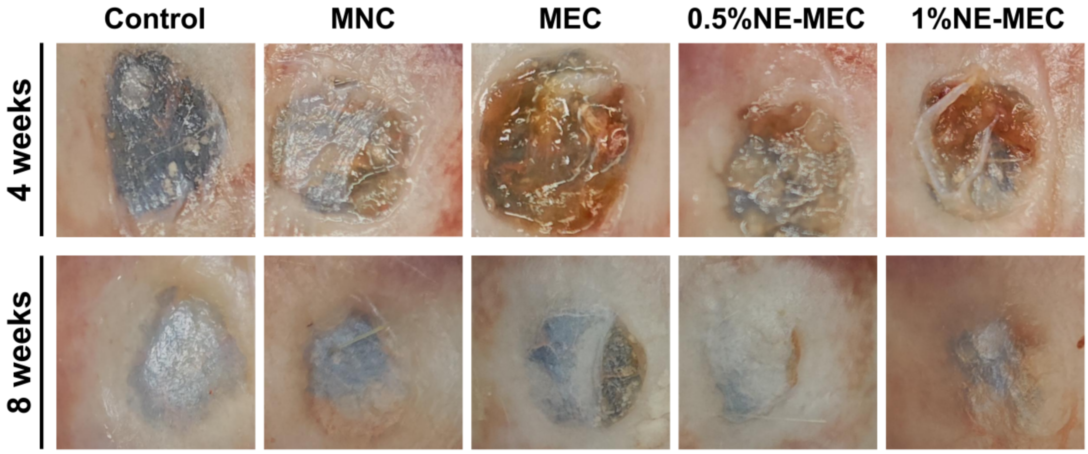

Supplementary Fig. 11| Representative macroscopic images showing in vivo degradation of implanted scaffolds at 4 and 8 weeks post-surgery.

Table S1. Primers sequences (for rat) used in the study.

Gene		Sequence (5’-3’)
Runx2	Forward	CATGGCCGGGAATGATGAG
	Reverse	TGTGAAGACCGTTATGGTCAAAGTG
Ocn	Forward	CCGTTTAGGGCATGTGTTGC
	Reverse	TTTCGAGGCAGAGAGAGGGA
Alp	Forward	TACTCGGACAATGAGATGCCGC
	Reverse	TTGTGCATTAGCTGATAGGCGA
Gdnf	Forward	TGTTCTCCTCTCCTGGCTGT
	Reverse	CTTCCTCCTCGAGTGTCGTG
Ngf	Forward	AAGGACGCAGCTTTCTATCC
	Reverse	CTATCTGTGTACGGTTCTGCC
Ntn4	Forward	CTGGGGCTTTGGAGACTACG
	Reverse	CGGGGACTTCGTGATACCAG
Wnt5β	Forward	CACTCAGGATCAGCGTGGGA
	Reverse	AGGAGTTGGCGTCAGTCAG
Runx1	Forward	GCTCCTTGGGGCATTTGACT
	Reverse	TGAGGTCGTTGAATCTCGCC
Spp1	Forward	TCAAGGTCATCCCAGTTGCC
	Reverse	GACTCATGGCTGGTCTTCCC
Tnf-a	Forward	ATCCGAGATGTGGAACTGGC
	Reverse	AAATGGCAAATCGGCTGACG
Vegf-c	Forward	TCATCAGCCAGGGAGTCTGT
	Reverse	GGGAGTGAAGGAGCAACCTC
Col1α1	Forward	CCAGCCGCAAAGAGTCTACAT
	Reverse	AGCACCATCGTTACCACGAG
Gapdh	Forward	ACAGCAACAGGGTGGTGGAC
	Reverse	TTTGAGGGTGCAGCGAACTT
